# Podophyllotoxin sensitizes triple-negative breast cancer cells to CD47-targeted immunotherapy

**DOI:** 10.1016/j.cellin.2026.100313

**Published:** 2026-03-13

**Authors:** Jessica Dang, Nuozi Song, Jingkai Zhou, Mustafa Raoof, Mingye Feng

**Affiliations:** a Department of Immuno-Oncology, Beckman Research Institute, City of Hope, Duarte, CA, 91010, USA; b Department of Surgery, City of Hope, Duarte, CA, 91010, USA; c Department of Cancer Genetics and Epigenetics, Beckman Research Institute, City of Hope, Duarte, CA, 91010, USA

## Abstract

Triple-negative breast cancer (TNBC) remains a highly aggressive malignancy with limited therapeutic options. Immunotherapeutic strategies are emerging as promising avenues to improve clinical outcomes in TNBC. Among these, CD47, a critical self-protective “don't eat me” immune checkpoint against macrophage immunosurveillance, is frequently upregulated in TNBC, contributing to tumor immune evasion. However, CD47 blockade alone has demonstrated limited efficacy in this context. To identify agents that potentiate CD47-targeted therapy, we conducted a high-throughput small molecule screen in TNBC models. This effort led to the identification of podophyllotoxin (PTOX), a plant-derived microtubule-disrupting agent, as a potent enhancer of macrophage-mediated TNBC clearance. PTOX treatment significantly sensitized TNBC cells to CD47 blockade-induced clearance. Gene set enrichment analysis (GSEA) revealed significant negative enrichment of PI3K_AKT signaling and positive enrichment of TNFα signaling via NF-κB in PTOX-treated TNBC cells. Functional perturbation studies further indicated that EGFR, TNFα, and TNFAIP3 contribute to PTOX-induced macrophage clearance. Together, these findings identify PTOX as a novel phagocytosis-sensitizing agent and support its potential as a combinatorial immunotherapeutic strategy to enhance CD47-targeted therapy in TNBC.

## Highlights

• High-throughput screening identifies PTOX as an immune-enhancing adjuvant in TNBC.
• PTOX sensitizes TNBC cells to macrophage-mediated phagocytosis.
• PTOX synergizes with CD47 blockade to suppress TNBC tumor growth *in vivo.*
• PTOX reprograms TNBC cells and reshapes the TME to favor PrCR.

## Graphical abstract

**Figure:**
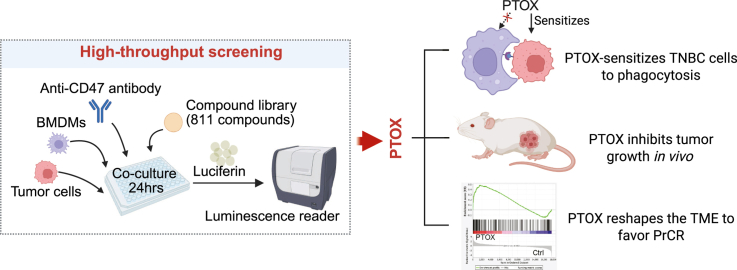

## 1 Introduction

Triple-negative breast cancer (TNBC) is among the most aggressive and therapeutically challenging subtypes of breast cancer, accounting for roughly 15% of all cases (Giaquinto et al., 2024; Popovic et al., 2023). To date, immune checkpoint inhibitors such as atezolizumab and pembrolizumab remain the only FDA-approved immunotherapeutic agents for TNBC, showcasing the potential of immunotherapy in this highly difficult-to-treat disease (Cortes et al., 2022; Mittendorf et al., 2025). However, clinical benefits remain limited, with only a small fraction of patients achieving meaningful responses. Progress is further hindered by the heterogeneity of TNBC, the absence of well-established molecular markers, and a high relapse rate driven by rapid tumor growth and acquired resistance (Asleh et al., 2022; Aysola et al., 2013; Ibrahim et al., 2024). As TNBC emerges as a leading cause of cancer-related mortality among women worldwide, there is an urgent clinical need for new treatment strategies.

Targeting the frequently immunosuppressive tumor microenvironment (TME) has emerged as a promising therapeutic approach. Modulation of the TME has shown considerable potential, either through the direct inhibition of tumor proliferation and survival or by inducing the tumoricidal roles of the immune cells (Eulberg et al., 2022; Giraldo et al., 2019; Kundu et al., 2024). Tumor-associated macrophages (TAMs) represent one of the most abundant immune populations within the TME of solid tumors, including TNBC (Hirano et al., 2023; Li et al., 2022; Sabit et al., 2025). Recent studies highlighted the pivotal role of macrophage-mediated immunosurveillance involving direct recognition and engulfment of cancer cells by macrophages, a process termed programmed cell removal (PrCR) (Banuelos et al., 2025; Chao et al., 2011; Li et al., 2024). To evade PrCR, various cancer cells, including TNBC, upregulate inhibitory “don't eat me” signals (Feng et al., 2019; Jaiswal et al., 2010). A central regulator of this process is CD47, an immune checkpoint binding to its cognate receptor SIRPα on macrophages, transmitting inhibitory signals to suppress PrCR (Chao et al., 2019; Logtenberg et al., 2020; Matlung et al., 2017). Notably, therapeutic blockade of the CD47-SIRPα interaction has demonstrated promising efficacy by restoring macrophage-mediated tumor clearance, thereby inhibiting tumor engraftment and preventing tumor growth (Willingham et al., 2012; Yuan et al., 2019; Zhao et al., 2022). CD47 has therefore emerged as a compelling therapeutic target across multiple cancer types, including both solid cancers and hematological malignancies (Jiang et al., 2021; Maute et al., 2022; Yang et al., 2023). However, clinical trials targeting CD47 have yielded mixed results. This is partly attributable to the complexity of the TME, the presence of compensatory immune-evasion mechanisms that diminish the effectiveness of CD47 blockade alone, and the dose limitations imposed by the toxicity associated with systemic CD47 inhibition. Consequently, there is a critical need to develop novel immunotherapeutic strategies capable of overcoming these limitations and improving treatment outcomes for TNBC. One promising approach is to identify anticancer small molecules or biologics to augment the efficacy of CD47-targeted therapy, thereby allowing lower doses of CD47-blocking agents and achieving more effective and durable therapeutic outcomes (Cao et al., 2021). Building on this concept, we conducted a high-throughput screen using a Mechanistic Set V compound library from the National Cancer Institute (NCI), a collection of compounds systematically profiled across the NCI-60 human tumor cell panel and enriched for agents with defined or putative mechanisms of action (Zweifach, 2020). The mechanistic diversity of this library provides a more direct interpretation of screening outcomes and facilitates the identification of pathway-level regulators of tumor cell susceptibility to phagocytosis. Through this screening, we identified podophyllotoxin (PTOX) as a promising adjuvant capable of markedly enhancing macrophage-mediated PrCR and improving antitumor immunity in TNBC. PTOX was originally utilized as a microtubule-targeting agent (MTA) to disrupt cancer cell division by inhibiting microtubule polymerization. While MTAs are among the most widely used chemotherapeutics, their efficacy as monotherapy is limited in achieving durable clinical responses. Increasing evidence indicates that MTAs also exert immunomodulatory effects, including reprogramming macrophages to an M1-like state with enhanced clearance of cancer cells (Cao et al., 2021; Wanderley et al., 2018). Microtubule disruption has been implicated in improving the efficacy of immune checkpoint inhibitors (Choi et al., 2024; Mantovani et al., 2017). These findings suggest that MTAs may complement checkpoint blockade therapy by not only suppressing tumor proliferation, migration, and invasion, but also by reshaping the TME. However, the direct effects of PTOX on TAMs and its potential as an immunomodulatory adjuvant to enhance PrCR in the TME remain unexplored.

Our results demonstrate that PTOX-treated TNBC cells become markedly more susceptible to macrophage-mediated phagocytosis. Importantly, combining PTOX with CD47 blockade leads to efficient elimination of TNBC cells. Notably, PTOX sensitizes tumor cells to PrCR at doses that do not directly induce apoptosis of the TNBC cells. In summary, our study identifies PTOX as a novel immunotherapeutic adjuvant that potentiates PrCR-based therapy, unveiling a new mechanistic avenue for the development of more effective immunotherapeutic strategies against TNBC.

## 2 Results

### 2.1 High-throughput screening identifies PTOX as an immune-enhancing adjuvant in TNBC

**Fig. 1:**
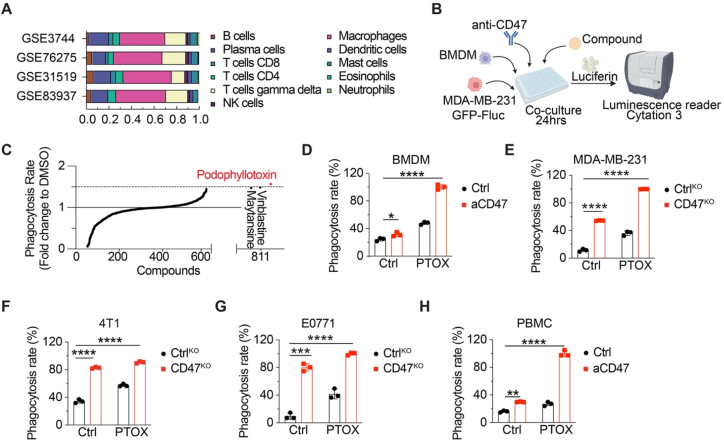
**High-throughput screening with NCI mechanistic diverse compounds identifies PTOX as a PrCR-mediating agent.** (**A**) Inferred composition of 11 immune subtypes of 4 TNBC biopsies, derived from the gene expression profiles available in the GEO database and analyzed using the CIBERSORT algorithm. (**B**) A schematic showing the high-throughput screening co-culturing macrophages, MDA-MB-231 cells, anti-CD47 blocking antibody and NCI compounds. (**C**) High-throughput screening analysis from luminescence-based assay. Phagocytosis was normalized to the DMSO control and the top 3 individual compounds Vincristine, PTOX, and Vinblastine were identified. (**D**) A luminescence-based *in vitro* phagocytosis assay displays the surviving cancer cells co-cultured with MDA-MB-231 cells. Mouse BMDMs were used, with cells treated with CD47-blocking antibody (1 μM). *n* = 3; two-way ANOVA with multiple comparison tests. (**E-G**) A luminescence-based *in vitro* phagocytosis assay showing surviving cancer cells co-cultured with MDA-MB-231 (E), 4T1 (F) and E0771 (G) cells. Each group was compared with the Ctrl group. *n* = 3; two-way ANOVA with multiple comparison tests. (**H**) A luminescence-based *in vitro* phagocytosis assay displays the surviving cancer cells co-cultured with MDA-MB-231 cells. Human PBMC-derived macrophages were used, with cells treated with CD47-blocking antibodies (1 μM). *n* = 3; two-way ANOVA with multiple comparison tests. ∗*P* < 0.05, ∗∗*P* < 0.01, ∗∗∗*P* < 0.001, ∗∗∗∗*P* < 0.000 1. Error bars represent ± SD.

PrCR-based therapies rely on leveraging macrophages within the TME to mediate the clearance of cancer cells. Quantitative analysis of the immune landscape in TNBC, utilizing RNA sequencing data from four cohorts of patient specimens, demonstrated TAMs as the most abundant immune cell population (Fig. 1(A)). To identify agents capable of boosting PrCR against TNBC cells, we conducted a high-throughput screen using the Mechanistic Set V compound library (811 compounds, obtained from the NCI). In this screen, macrophages were co-cultured with a TNBC line, MDA-MB-231, in the presence of a CD47-blocking antibody and the individual compounds in the library (Fig. 1(B)). MDA-MB-231 cells were engineered to express a GFP-luciferase reporter, enabling a quantified assessment of TNBC cell survival. Luminescence signals from compound-only treatment were used to normalize the results and determine the efficacy of PrCR. Among the compounds screened, PTOX emerged as one of the most potent enhancers of PrCR (Fig. 1(C)). PTOX is a plant-derived natural product first isolated from *Podophyllum peltatum L*. It acts as an antineoplastic agent, functioning by binding to tubulin and destabilizing microtubule polymerization, ultimately inhibiting cell division. Previous studies have demonstrated PTOX and its derivatives, such as etoposide and teniposide, inhibit cancer cell proliferation as well as suppress migration and invasion (Fan et al., 2021; Motyka et al., 2023). Although PTOX is associated with dose-dependent cytotoxicity, its strong antitumor activity supports its potential as a promising candidate for combination therapies. We hypothesize that if PTOX and CD47-blocking agents can exert synergistic anticancer effects when administered concurrently, both agents could be employed at reduced doses to mitigate potential toxicity.

To validate our screen results, we assessed the effects of PTOX at varying concentrations on both human and murine TNBC cell lines (Fig. S1A–E) using FACS-based 2h phagocytosis assays. PrCR was strongly induced at concentrations as low as 0.3 μM PTOX, at which minimal direct cytotoxicity was detected across all TNBC cell lines (Fig. S1A–E). This finding suggests the potential utility of PTOX as an immune adjuvant at subtoxic doses, minimizing toxicity to normal tissue cells. Based on these findings, we selected 1 μM PTOX as a subtoxic yet effective dose for further investigation into its immune-enhancing properties (Fig. S1A–E). To validate the role of PTOX in promoting PrCR, we employed two CD47-blockade models, (1) a blocking antibody to disrupt CD47-SIRPα interactions, and (2) genetic suppression of CD47 in TNBC cells. First, using luminescence-based phagocytosis assays quantifying surviving cancer cells upon coculturing with macrophages against TNBC cells, we found CD47 blockade alone promoted phagocytosis of MDA-MB-231 cells, while the combination with PTOX significantly enhanced this effect (Fig. 1(D)). Similarly, CD47 knockout (CD47^KO^) in MDA-MB-231, 4T1 or E0771 TNBC cells using CRISPR-mediated gene editing led to increased PrCR, which was further amplified by the addition of PTOX (Fig. 1(E–G) and Fig. S1F–G). Notably, PTOX treatment itself did not change CD47 expression (Fig. S1H). Consistent results were observed using human peripheral blood monocyte-derived macrophages (PBMC), where PTOX synergized with CD47 blockade to significantly enhance PrCR (Fig. 1(H)). These results indicate that PTOX, at subtoxic doses, efficiently enhances macrophage-mediated phagocytosis against TNBC cells.

### 2.2 PTOX sensitizes TNBC cells to macrophage-mediated PrCR without inducing cytotoxicity

**Fig. 2:**
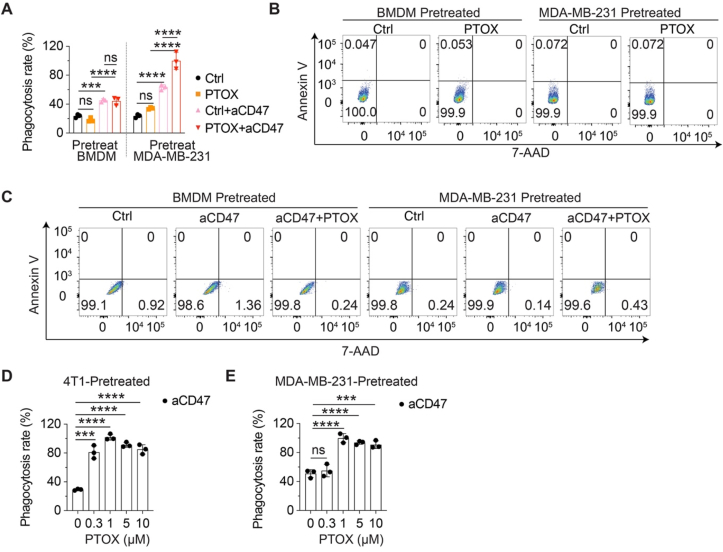
**PTOX enhanced PrCR by sensitizing TNBC cells susceptibility to the recognition and elimination by macrophages**. (**A**) A luminescence-based phagocytosis assay of BMDMs and MDA-MB-231 cells co-cultured in the presence of CD47-blocking antibody. Cells were either untreated or pretreated with PTOX. *n* = 3; two-way ANOVA with multiple comparison tests. (**B-C**) Representative FACS images of Annexin V/7-AAD staining of MDA-MB-231 and BMDMs pretreated with PTOX. (**D**) Flow cytometry-based phagocytosis assay of BMDMs and 4T1 cells co-cultured for 2 h. Cells were either untreated or pretreated with PTOX. *n* = 3; two-way ANOVA with multiple comparison tests. (**E**) A luminescence-based phagocytosis assay of MDA-MB-231 cells and BMDMs co-cultured for 24 h in the presence of CD47-blocking antibody. TNBC cells were pretreated with PTOX. *n* = 3; two-way ANOVA with multiple comparison tests. ns, no significance, ∗∗∗*P* < 0.001, ∗∗∗∗*P* < 0.000 1. Error bars represent ± SD.

We reasoned PTOX is functioning as a potentiator of PrCR by either enhancing the phagocytic capability of macrophages or by increasing the susceptibility of tumor cells to phagocytosis. To test this, we performed a set of co-culture experiments in which either macrophages or TNBC cells were pretreated with PTOX prior to co-culture with untreated counterpart cells: (1) BMDM were pretreated with PTOX and then co-cultured with untreated MDA-MB-231 cells, or (2) MDA-MB-231 cells were pretreated with PTOX and co-cultured with untreated BMDMs. We found that PTOX-pretreated BMDMs exhibited only a modest increase in PrCR, whereas PTOX-pretreated MDA-MB-231 cells demonstrated a significant enhancement in PrCR (Fig. 2(A)). These findings suggested PTOX primarily augments PrCR by sensitizing TNBC cells, thereby increasing their susceptibility to macrophage-mediated recognition and elimination. To further evaluate whether this enhanced PrCR resulted from cytotoxic effects of PTOX, we assessed the viability of MDA-MB-231 cells treated with 1 μM PTOX using Annexin V/7-AAD staining. The analysis showed no discernible reduction in viability compared with vehicle-treated controls, indicating PTOX-mediated enhancement of PrCR occurs independent of direct cytotoxicity (Fig. 2(B) and (C)).

We then extended our investigation to additional TNBC lines, including 4T1, E0771, and MDA-MB-468 cells. Consistent with the observations in MDA-MB-231 cells, pretreatment of TNBC cells with PTOX led to a robust increase in phagocytosis (Fig. 2(D–E) and Fig. S2A–D) using both luminescence-based phagocytosis assays and FACS-based assays. Collectively, these data demonstrated that PTOX sensitizes TNBC cells to macrophage-mediated phagocytosis through a mechanism independent of direct cytotoxicity.

### 2.3 PTOX synergizes with CD47 blockade to suppress tumor growth across diverse TNBC models

**Fig. 3:**
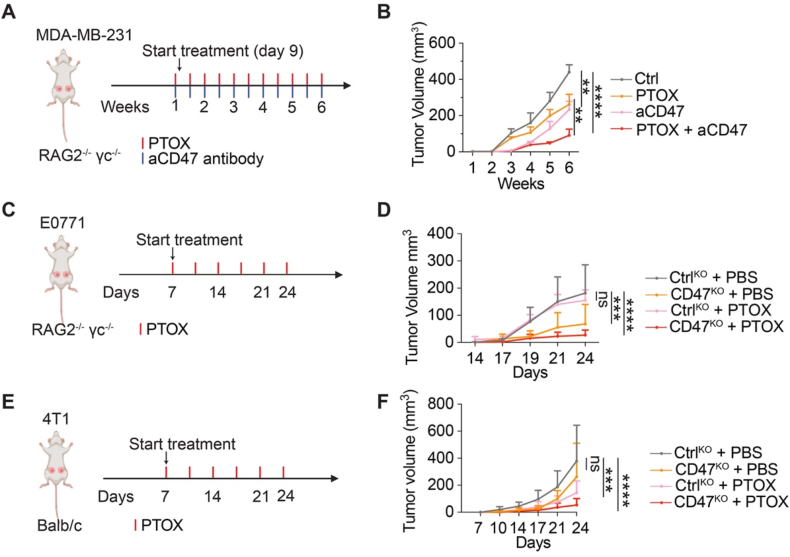
**PTOX enhances the therapeutic efficacy in combination with CD47 blockade inhibiting the progression of TNBC cells.** (**A**) A schematic diagram of the treatment regimen for MDA-MB-231 cells in RAG2^−/−^ γc^−/−^ mice. Mice were treated orthotopically with PBS, anti-CD47 antibody, PTOX or combination of anti-CD47 antibody and PTOX. (**B**) Tumor growth was measured using a caliper. *n* = 6 mice; two-way ANOVA with multiple comparison tests. (**C**) A schematic diagram of the treatment of E0771 cells in RAG2^−/−^ γc^−/−^ mice. Mice were treated orthotopically with PBS or PTOX. (**D**) Tumor growth was measured using a caliper. *n* = 4 mice; two-way ANOVA with multiple comparison tests. (**E**) A schematic diagram of the treatment of PTOX for 4T1 Ctrl^KO^ or CD47^KO^ cells in BALB/c mice. Mice were treated orthotopically with PBS or PTOX. (**F**) Tumor growth was measured using a caliper. *n* = 7 or 8 mice; two-way ANOVA with multiple comparison tests. ns, no significance, ∗∗*P* < 0.01, ∗∗∗*P* < 0.001, ∗∗∗∗*P* < 0.000 1. Error bars represent ± SD.

Building on our *in vitro* findings that PTOX sensitizes TNBC cells to macrophage-mediated PrCR, we next evaluated its *in vivo* therapeutic potential in combination with CD47 blockade using three TNBC mouse models differing in cell type, CD47-targeting strategies, and immune context. First, in a human xenograft model, MDA-MB-231 cells were orthotopically implanted into the mammary fat pads of Rag2^−/−^ γc^−/−^ mice, which lack T, B, and NK cells but retain functional myeloid compartments. On day 9, mice were treated with vehicle control, PTOX, CD47-blocking antibody, or the combination of both (Fig. 3(A)). While monotherapy with either CD47-blocking antibody or PTOX moderately suppressed tumor growth, combination therapy dramatically inhibited tumor progression, indicating a strong synergistic antitumor effect (Fig. 3(B)). Next, to assess whether this synergy extends to murine TNBC tumors, E0771 cells were orthotopically implanted into the mammary fat pads of Rag2^−/−^ γc^−/−^ mice. In this model, Ctrl^KO^ and CD47^KO^ cells were generated via transduction with Cas9 and either a control sgRNA (non-targeting) or a CD47-targeting sgRNA, respectively. Mice bearing Ctrl^KO^ or CD47^KO^ tumors received PTOX starting on day 7 after tumor inoculation (Fig. 3(C)). PTOX treatment markedly reduced tumor volume in CD47^KO^ tumors compared with all other groups, including PTOX-treated Ctrl^KO^ tumors, further supporting a cooperative effect between PTOX treatment and CD47 deficiency (Fig. 3(D)). Lastly, to assess the efficacy of PTOX-CD47-blockade in an immunocompetent setting, we utilized a 4T1 syngeneic TNBC model in BALB/c mice. Ctrl^KO^ and CD47^KO^ cells were orthotopically implanted into the mammary fat pads, and mice received either PTOX or vehicle control beginning on day 7 post-inoculation (Fig. 3(E)). Consistent with the prior models, PTOX significantly suppressed tumor growth in the CD47^KO^ group (Fig. 3(F)). Collectively, these results demonstrated that PTOX consistently enhances the antitumor effects of CD47 blockade across multiple TNBC models, supporting its potential as a potent adjuvant for macrophage-based cancer immunotherapy.

### 2.4 PTOX reprograms TNBC cells and reshapes the tumor immune microenvironment to favor PrCR

**Fig. 4:**
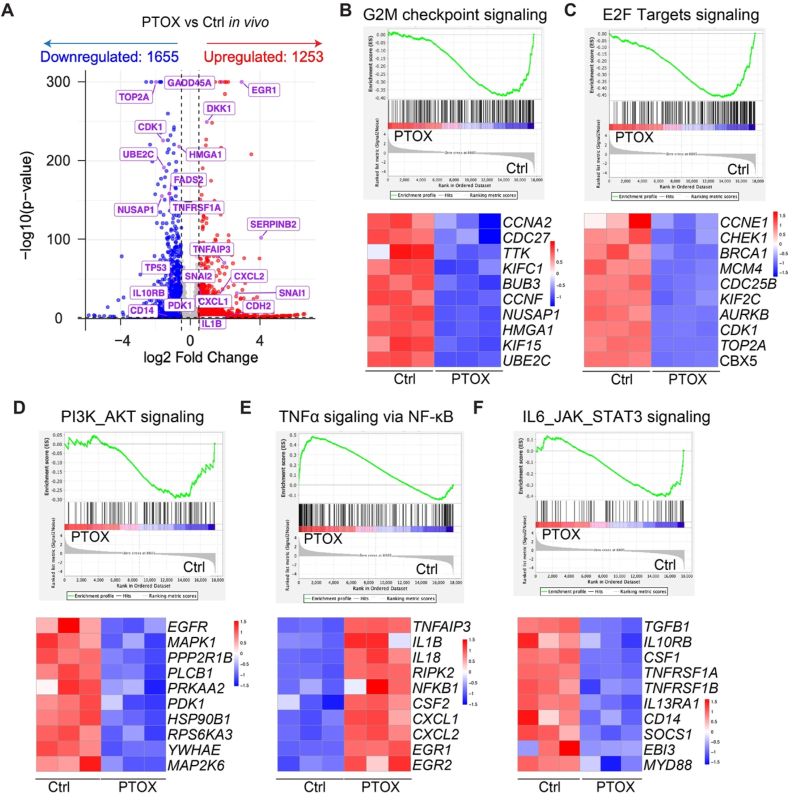
**Bulk RNA sequence analysis reveals PTOX-induced transcriptional reprogramming and pathways alterations.** (**A**) A volcano plot showing differentially expressed genes (DEGs) in DMSO-treated and PTOX-treated MDA-MB-231 cells. Genes with *p* value < 0.01 and fold change > 1.5 were considered significantly upregulated or downregulated in bulk RNA sequencing (*n* = 3). (**B-F**) GSEA of tumor samples from PTOX-versus Ctrl-treated groups revealed significant changes in key signaling pathways. Enrichment plots (top) show pathway enrichment scores, with corresponding heatmaps (bottom) depicting representative genes differentially expressed between PTOX and Ctrl group.

To further investigate how PTOX reprograms TNBC cells to become more susceptible to PrCR, we performed bulk RNA sequencing of MDA-MB-231 cells treated with either vehicle control or PTOX for 24 h. Transcriptomic profiling revealed a distinct gene expression landscape in PTOX-treated cells, with 1 253 genes significantly upregulated and 1 655 genes downregulated (Fig. 4(A)). Gene set enrichment analysis (GSEA) revealed pronounced downregulation of the G2/M checkpoint signaling (*CCNA2, CDC27*, *TTK*, *KIFC1*, *BUB3*, *KIF15*, *UBE2C*, *CCNF*, *NUSAP1*, *HMGA1*) (Fig. 4(B)) and E2F targets signaling (*CCNE1*, *CHEK1*, *BRCA1*, *MCM4*, *CDC25B*, *KIF2C*, *AURKB*, *CDK1*, *TOP2A*, *CBX5*) (Fig. 4(C)), signatures associated with tumor cell proliferation (Chen et al., 2025). Similarly, gene signatures linked to proliferative and stress-responsive PI3K_AKT signaling were downregulated, including *EGFR, MAPK1*, *PPP2R1B*, *PLCB1*, *PRKAA2*, *PDK1*, *HSP90B1*, *RPS6KA3*, *YWHAE*, *MAP2K6* (Fig. 4(D)). Notably, tumor necrosis factor alpha (TNF⍺) signaling via NF-κB was strongly enriched after PTOX treatment, accompanied by upregulation of multiple inflammatory cytokines and immune-related genes (*TNFAIP3*, *IL-1B*, *IL-18*, *RIPK2*, *NF-KB1*, *CSF2*, *CXCL1*, *CXCL2*, *EGR1*, *EGR2*) (Fig. 4(E)). In parallel, the IL-6_JAK1_STAT3 signaling (*TGFB1*, *IL1*0RB, *CSF1*, *TNFRSF1A*, *TNFRSF1B*, *IL13RA1*, *CD14*, *SOCS1*, *EBI3*, *MYD88*) (Fig. 4(F)) was downregulated. This pathway in tumor cells is known to drive immunosuppression through MDSC recruitment and macrophage polarization; therefore, its inhibition may contribute to reshaping TME (Jing et al., 2020; Jones et al., 2016; Lee et al., 2011). Together, these results indicate that PTOX broadly suppresses proliferative and immune-evasive programs while activating TNFα-driven inflammatory signaling, thereby reprogramming TNBC cells into a more immunologically visible state to favor macrophage-mediated PrCR.

### 2.5 Inflammatory-associated signaling pathways contribute to PTOX-induced sensitization of TNBC cells to PrCR

**Fig. 5:**
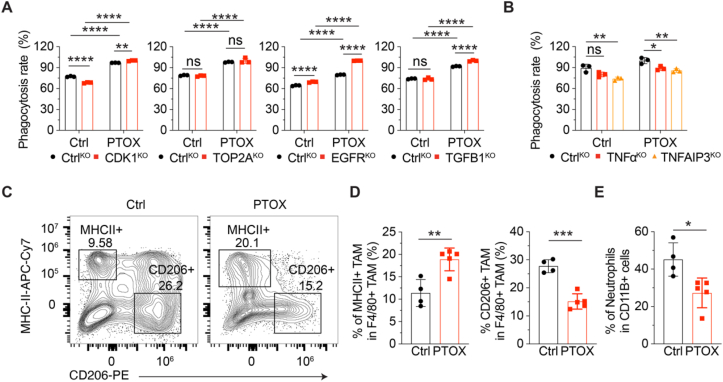
**EGFR and TNFα contribute to PTOX-induced PrCR sensitization in TNBC cells.** (**A-B**) A luminescence-based phagocytosis assay with MDA-MB-231 cells as target cells with Ctrl^KO^, CDK1^KO^, TOP2A^KO^, EGFR^KO^, TGFB1^KO^, TNFα^KO^, and TNFAIP3^KO^. In all assays, mouse BMDMs were used for the assay. Cells were treated with CD47-blocking antibody and PTOX with 1 μM. *n* = 3 mice; two-way ANOVA with multiple comparison tests. (**C-D**) Representative FACS plots of TAMs and quantification of MHCII+ or CD206+ TAMs among F4/80+ TAMs from MDA-MB-231 tumor cells. *n* = 4 (Ctrl) or 5 (PTOX) mice; unpaired *t*-test. (**E**) Quantification of Neutrophils among CD11B+ cells from MDA-MB-231 tumor cells. *n* = 4 (Ctrl) or 5 (PTOX) mice; unpaired *t*-test. ∗*P* < 0.05, ∗∗*P* < 0.01, ∗∗∗*P* < 0.001, ∗∗∗∗*P* < 0.000 1. Error bars represent ± SD.

To functionally validate the contribution of these signaling pathways to PTOX-mediated PrCR, we performed loss-of-function assays targeting representative genes within the regulatory networks and examined their effects on macrophage phagocytosis. Knockout of CDK1 or TOP2A, two canonical mitotic regulators, had minimal impact on PTOX-induced phagocytosis (Fig. 5(A)), suggesting that suppression of the CDK1 and TOP2A reflects a transcriptional consequence rather than a direct mechanism driving the phagocytic response. In contrast, deletion of EGFR or TGFB1, both of which were downregulated after PTOX treatment (Fig. 4(D) and (F)), further enhanced PTOX-induced phagocytosis (Fig. 5(A)). These findings are consistent with prior reports indicating that EGFR signaling may modulate phagocytosis checkpoints and inhibition of TGF-β signaling facilitates macrophage activation (Hu et al., 2024; Massagué, 2008; Wrzesinski et al., 2007). Together, these results suggest PTOX-induced sensitization is associated with suppression of EGFR- and TGF-β-related transcriptional programs, rather than regulation of classical proliferation-associated genes (CDK1 and TOP2A). TNFα is a central upstream regulator of pro-inflammatory cytokine cascades that drive macrophage polarization toward a pro-inflammatory M1-like phenotype (Chen et al., 2023). In line with enriched TNFα signaling and elevated TNFα secretion after PTOX treatment (Fig. 4(E) and Fig. S3A), knockout of TNFα or its downstream effector TNFAIP3 significantly attenuated the PTOX-induced enhancement of PrCR (Fig. 5(B) and Fig. S3A), supporting a mechanistic link between PTOX-induced TNF⍺ signaling and increased tumor cell susceptibility to macrophage phagocytosis. Collectively, these results demonstrate that PTOX reprograms TNBC cells at the transcriptional level to enhance their immunogenicity and susceptibility to PrCR, in part through activation of inflammation-associated signaling pathways.

To determine whether these transcriptional changes in TNBC cells translate into remodeling of the TME *in vivo*, we implanted MDA-MB-231 cells into the mammary fat pads of Rag2^−/−^ γc^−/−^ mice and treated them with PTOX. Flow cytometry analysis of tumor-infiltrating immune cells revealed that PTOX-treated tumors exhibited a marked increase in the ratio of MHCII+ TAMs and a concomitant decrease in CD206+ TAMs (Fig. 5(C–D) and Fig. S3B), indicating a shift of TAMs toward a pro-inflammatory, M1-like phenotype. Moreover, PTOX treatment significantly reduced the proportion of tumor-associated neutrophils (Fig. 5(E)), a population known to promote anti-inflammatory macrophage polarization (Marwick et al., 2018). Together, these findings suggest that PTOX not only reprograms TNBC cells to adopt a more pro-inflammatory and immunogenic phenotype but also remodels the tumor immune microenvironment to favor effective PrCR.

## 3 Discussion

Immunotherapeutic options for TNBC remain limited, with only modest improvements in overall survival and disease-free progression. Current FDA-approved options include immune checkpoint inhibitors such as atezolizumab and pembrolizumab, but their benefits are largely restricted to patients with PD-L1-positive tumors. For instance, an ongoing phase III trial KEYNOTE-522 (NCT03036488) reported that neoadjuvant pembrolizumab with paclitaxel and carboplatin resulted in a significant improvement in overall survival by a modest 5%, however, this treatment only yields success for select patients with PD-L1+ TNBC expression (Schmid et al., 2024). Similarly, ASCENT-04/KEYNOTE-D19 (NCT0582286) using pembrolizumab with sacituzumab govitecan-hziy on patients with advanced TNBC, whose tumors expressed PD-L1, demonstrated superior disease-free progression compared to chemotherapy plus pembrolizumab (Tolaney et al., 2025). These results suggest these treatments rely on PD-L1 as a predictive biomarker to successful progressive survival. However, the diverse molecular and immunological landscape of TNBC likely contributes to differential treatment outcomes, underscoring the need for effective therapeutic approaches for all TNBC subgroups.

One approach to overcome treatment-resistance involves targeting additional immune evasion mechanisms or effectors such as TAMs (Cassetta & Pollard, 2018; Jalil et al., 2020; Willingham et al., 2012). Given the upregulation of CD47 and the dense macrophage infiltration observed in TNBC tumors, PrCR-based therapy represents a promising new class of immunotherapy for TNBC(Chen et al., 2022; Mediratta et al., 2020). Importantly, PrCR harnesses TAMs that are abundant in TME, and its mechanism of action is distinct from other immune checkpoint pathways. Therefore, activating PrCR may provide an effective therapeutic strategy complementary to existing immune checkpoint inhibitors (Li et al., 2024). In this study, we demonstrated that despite its traditional classification as an anti-tumor cytotoxic compound with limited clinical use due to toxicity, PTOX at subtoxic concentrations acts as a strong immunomodulatory agent that sensitizes TNBC cells to PrCR-mediated phagocytosis. PTOX synergizes with CD47-blocking antibodies to markedly enhance phagocytosis independent of cellular cytotoxicity and tubulin disruption *in vitro* and suppress tumor growth *in vivo*. Mechanistically, our findings indicate PTOX enhances PrCR primarily by sensitizing tumor cells rather than directly activating macrophage-intrinsic phagocytic programs. This conclusion is supported by pretreatment experiments showing that PTOX-treated TNBC cells exhibited a marked increase in phagocytosis, whereas PTOX pretreatment of macrophages resulted in only a modest effect. This demonstrates that PTOX's immune enhancing activity is largely mediated by tumor cell reprogramming rather than direct activation of macrophage intrinsic phagocytic capabilities. At the molecular level, PTOX reshapes the transcriptional landscape of tumor cells by downregulating pathways associated with immune evasion (PI3K_AKT and IL-6_JAK_STAT3 signaling) while upregulating pro-inflammatory TNFα signaling. This transcriptional reprogramming weakens the tumor cells' ability to maintain an “immune-evasive” state and lowers the threshold for phagocytic engagement, effectively shifting tumor cells from a phagocytosis-resistant to a phagocytosis-permissive state. Collectively, these findings support a model in which PTOX functions predominantly as a phagocytosis-sensitizing agent rather than a direct activator of macrophage-intrinsic phagocytic machinery.

Transcriptomic profiling revealed that PTOX treatment induced extensive transcriptional reprogramming *in vivo*, characterized by suppression of proliferation-associated pathways, including the G2/M checkpoint, E2F targets, and PI3K_AKT signaling, alongside enrichment of inflammatory-related pathways TNFα signaling via NF-κB and downregulated stress-related signaling IL-6_JAK_STAT3. These results suggest that PTOX reprograms TNBC cells into a more immunologically visible state, thereby favoring macrophage-mediated PrCR. Although PTOX downregulates proliferative pathways, the enhanced phagocytosis is unlikely to result merely from growth inhibition. Both short-term and long-term phagocytosis assays consistently demonstrated increased CD47-mediated clearance without detectable changes in proliferation or apoptosis. The knockout of CDK1 or TOP2A had minimal impact on PTOX-induced phagocytosis suggesting that while PTOX, as a microtubule inhibitor, indeed suppresses G2/M and E2F signaling, the downregulation of these pathways-related genes does not directly promote phagocytosis through reduced proliferation. Instead, it likely reflects a broader shift in cellular state that renders tumor cells more susceptible to PrCR. Notably, G2/M checkpoint activation has been associated with the formation of M2 macrophage-dominant immune barriers and resistance to immunotherapy (Chen et al., 2025). The E2F transcription factors, beyond their classical roles in G1/S regulation, have been implicated in angiogenesis, metabolic reprogramming, apoptosis resistance, and metastasis (Chen et al., 2009; Hallstrom et al., 2008; Phillips & Vousden, 2001; Sears & Nevins, 2002; Weijts et al., 2018). Recent studies have also revealed low E2F2 activity is associated with genomic instability, and E2F2 expression correlates with response to PARP inhibition therapy in breast cancer (Rennhack & Andrechek, 2020). In our study, PTOX shifted macrophage polarization from CD206+ immunosuppressive phenotype toward an MHCII+ M1-like phenotype. Moreover, PTOX reshapes the inflammatory signaling landscape by upregulating TNFα signaling via NF-κB and downregulating IL-6_JAK_STAT3 pathways, favoring the development of a pro-inflammatory M1-like macrophage phenotype (Fu et al., 2017; Xiang et al., 2021; Zhao et al., 2023). Functionally, deletion of TNFα or its regulator TNFAIP3 abrogated PTOX-induced phagocytosis, confirming this axis as essential for PTOX's immune-activating effect. Collectively, these findings demonstrate that PTOX enhances macrophage-mediated clearance through coordinated transcriptional and inflammatory reprogramming. By providing an inflammatory context that CD47 blockade alone cannot achieve, PTOX effectively sensitizes TNBC cells to phagocytic elimination and reshapes the tumor-immune interface toward an immune-permissive state.

PTOX is a potent antimitotic agent mediated through tubulin binding, resulting in mitotic arrest and cell death; its direct systemic application as an anticancer agent alone is precluded by significant toxicity and a narrow therapeutic window (Motyka et al., 2023; Zhao et al., 2021). These limitations historically drove the development of semisynthetic derivatives such as etoposide and teniposide, which retain antitumor efficacy through stabilization of topoisomerase II-DNA cleavage complexes and are now widely used in clinical oncology, albeit with myelosuppression and resistance (Neupane et al., 2011; Zhao et al., 2021). Recent formulation efforts, including prodrug strategies and nanocarrier-based delivery systems, aim to improve PTOX bioavailability and tumor selectivity while mitigating systemic toxicity, thereby renewing interest in this agent for translational development (Miranda-Vera et al., 2025; Wang et al., 2016). Importantly, our findings redefine PTOX not solely as a cytotoxic agent but as a tumor-directed immune sensitizer. The phagocytosis-enhancing activity was observed under conditions that do not rely on maximal cytotoxicity, suggesting that clinically meaningful immune modulation may be achievable at lower, potentially less toxic doses. In combination with anti-CD47 blockade, PTOX may serve to prime tumor cells for macrophage-mediated clearance, thereby allowing dose reduction while preserving therapeutic efficacy. This approach provides a rational framework for integrating PTOX or optimized derivatives into macrophage-directed immunotherapy regimens.

Together, these results support the model in which PTOX enhances PrCR through the integrated activation of proliferation-related stress and inflammation-associated pathways, functioning not merely as a cytotoxic agent but as an immunomodulatory sensitizer. Further exploration into how PTOX interacts with these signaling networks may uncover new combinatorial strategies to amplify the innate immune clearance in aggressive breast cancer.

## 4 Materials and methods

### 4.1 Cell lines

The experiments included human triple-negative breast cancer cell lines MDA-MB-231 and MDA-MB-468, and mouse triple-negative breast cancer cell lines E0771 and 4T1. All cell lines were purchased from the American Type Culture Collection (ATCC). MDA-MB-231, MDA-MB-468, and E0771 cells were maintained in Dulbecco's Modified Eagle Medium (DMEM) supplemented with 10% fetal bovine serum and 1% penicillin/streptomycin, while 4T1 cells were cultured in RPMI-1640 supplemented with 10% fetal bovine serum (FBS) and 1% penicillin/streptomycin. All cell lines were maintained at 37 °C in a humidified atmosphere containing 5% CO_2_. To ensure experimental consistency, large batches of cells from passages below three were cryopreserved, and only cells with passage numbers under 20 were used throughout the study. Mycoplasma examination was routinely performed.

### 4.2 Mice

A series of murine models were used in the experiments, including BALB/c, C57BL/6, and Rag2 ^−/−^ γc^−/−^ mice. The BALB/c and C57BL/6 mouse strains were purchased from the Jackson Laboratory. The Rag2 ^−/−^ γc^−/−^ strain was a generous gift from Dr. Irvin L. Weissman at Stanford University. All mice were bred and maintained in the Animal Resources Center at City of Hope Comprehensive Cancer Center, registered by the United States Department of Agriculture and accredited by the Association for Assessment and Accreditation of Laboratory Animal Care International (AAALAC).

All animal procedures were conducted in accordance with guidelines and approved by the Administrative Panel on the Laboratory Animal Care at the City of Hope Comprehensive Cancer Center. Mice were housed under controlled conditions: 12-h light/dark cycle, temperatures 18-23 °C, and relative air humidity 40%-60%. Prior to cancer-related experiments, all mice were confirmed to be in good health and assessed as BAR (bright, alert, and responsive). Health status was monitored daily. Female mice aged 8 to 12 weeks were used for all the experiments.

### 4.3 Generation of primary macrophages

Mouse BMDMs were generated from 6 to 12-weeks-old BALB/c or C57BL/6 mice. Following euthanasia, the femurs were extracted, and bone marrow cells were passed through a 70 μm strainer. The cells were pelleted by the centrifuge and treated with ACK lysis buffer at room temperature for 5 min to remove red blood cells, then cultured with IMDM supplements with 10% FBS and 10 ng/mL murine M-CSF for 6-8 days to stimulate macrophage differentiation.

Human peripheral blood-derived macrophages were generated by isolating monocytes from the buffy coat obtained through leukapheresis cones by Ficoll-Paque density gradient centrifugation. The monocytes were enriched from human PBMCs by using CD14-positive selection beads (Miltenyi Biotec) and cultured with IMDM supplemented with 10% human serum for 7-10 days to allow differentiation into macrophages.

### 4.4 High-throughput screening

The Mechanistic Set V, consisting of 811 compounds derived from the 37 836 open compounds tested in the NCI human tumor 60 cell line screen, was obtained from the Division of Cancer Treatment & Diagnosis at the NCI (Supplemental Table 1). For the high-throughput screening, MDA-MB-231 cells expressing GFP-luciferase (0.03 × 10^6^ cells) were co-cultured with macrophage (0.06 × 10^6^ cells) in the presence of CD47 blockades in 96-well plates for 24 h. Thereafter, luciferin was added into the plate, and a luminescence signal was measured with Cytation 3. The wells containing 1 μM drug and MDA-MB-231 cells only were used as a control for calculating phagocytosis rate (phagocytosis rate = 0%). The normalized phagocytosis index was calculated as the ratio of phagocytosis rate induced by drug treatment to that by DMSO.

### 4.5 Analysis of clinical dataset

For the analysis of the immune composition in TNBC tumors, three independent studies were included in the analysis. Gene expression datasets of these studies (GSE3744, GSE76275, GSE31519, and GSE83937) were obtained from the Gene Expression Omnibus (GEO) (https://www.ncbi.nlm.nih.gov/geo/). The analytical tool CIBERSORT (https://cibersort.stanford.edu/) was used to estimate the relative proportion of the composition of immune cell subsets in TNBC biopsies.

### 4.6 CRISPR/Cas9-mediated gene editing

The CRISPR/Cas9 system was used to knock down gene expression in TNBC cells. Control sgRNAs, as well as sgRNAs for targeting human CD47, mouse CD47, human CDK1, human TOP2A, human EGFR, human TGFB1, human TNFAIP3, and human TNFα were designed and cloned into the all-in-one Lenti-CRISPR V2 vector. The LentiCRISPR V2 vector, together with packaging plasmids, was transfected into 293T cells to generate lentivirus. Viral supernatants were collected 48h post-transfection and filtered through a 0.45 μm filter to remove residual cells and debris. For transduction, target cells were incubated with lentiviruses for 48 h in the presence of polybrene (8 μg/mL). Following virus removal, cells were selected with blasticidin (20 μg/mL) or puromycin (2 μg/mL) for 24-72 h to enrich for successfully transduced populations.

The following sgRNA sequences were used:

Control: GAACGUAGAAAUUCCCAUUU.

Human CD47: CUACUGAAGUAUACGUAAAG.

Mouse CD47: CCCUUGCAUCGUCCGUAAUG.

Human CDK1: GGGUUCCUAGUACUGCAAUU.

Human TOP2A: UUCUGUGGAAUUAGUGACCC.

Human EGFR: UGAGCUUGUUACUCGUGCCU.

Human TGFB1: CGGGUUCAGGUACCGCUUCU.

Human TNFα: CGAUCACUCCAAAGUGCAGC.

Human TNFAIP3: CGGGGCUUUGCUAUGAUACU.

### 4.7 Phagocytosis assay

Macrophage-based phagocytosis was assessed using either luminescence- or flow cytometry-based assays. For long-term assays (24 h), macrophages were harvested using TrypLE and co-cultured with GFP-expressing target cancer cells at 37 °C in the presence of anti-CD47 antibody, an IgG control (BioLegend), and PTOX. The human anti-CD47 clone B6H12 (BioXcell) was used for blocking CD47 on human TNBC cells. Wells containing cancer cells alone, with or without PTOX, served as controls. Following the incubation period, D-Luciferin was added, and the luminescence signals were measured using the Biotek Cytation 3 to quantify viable cancer cells. The phagocytosis rate was calculated as the ratio of signals in the treatment group to the signal of wells containing cancer cells alone. The phagocytosis index was normalized to the maximal response to the experiment.

For the pre-treatment experiments, either cancer cells or macrophages were pre-treated with various concentrations of PTOX, with or without B6H12, for 24 h prior to co-culture. Luminescence was then measured as described above. Phagocytosis index was normalized to the maximal response in each experiment.

For the short-term assay (2 h), target cancer cells or macrophages were pre-treated with various concentrations of PTOX for 24 h, then co-cultured with untreated counterparts at 37 °C in the presence of anti-CD47 antibody. Phagocytosis assay was assessed by flow cytometry using a BD LSR Fortessa (BD Biosciences), and phagocytosis index was normalized to the maximal response in each experiment.

### 4.8 Mouse models

In the human TNBC model, MDA-MB-231 cells stably expressing a luciferase-GFP fusion protein were suspended in RPMI medium containing 25% Corning Matrigel Matrix (*V*/*V*) and injected into the mammary fat pad of the RAG2^−/−^, γc^−/−^ mice at 5 × 10^5^ cells per mouse. Nine days after engraftment, mice received an intratumoral injection of vehicle (Control) twice a week, CD47-blocking antibody (clone B6H12; 1 mg/kg body weight), PTOX (40 μg/kg body weight) or a combination of both.

In the mouse TNBC model, Ctrl^KO^ and CD47^KO^ E0771 cells expressing a luciferase-GFP fusion protein were suspended in RPMI medium containing 25% Corning Matrigel Matrix (*V*/*V*) and injected into the mammary fat pad of the RAG2^−/−^, γc^−/−^ mice at 1 × 10^5^ cells per mouse. Seven days after engraftment, the mice were treated with vehicle or PTOX (40 μg/kg body weight) intratumorally, twice every week.

In the syngeneic immunocompetent model, Ctrl^KO^ and CD47^KO^ 4T1 cells were suspended in RPMI medium containing 25% Corning Matrigel Matrix (*V*/*V*) and injected into the mammary fat pad of the BALB/c mice with 5 × 10^5^ cells per mouse. Seven days after the engraftment, the mice were treated with vehicle (Control) or PTOX (40 μg/kg body weight), twice every week.

Tumor size was determined by measuring the length (*L*) and width (*W*) of each tumor with calipers, and tumor volume was calculated using formula (*L* × *W* × *W*)/2. All tumors were monitored to ensure their maximal diameter did not exceed 15 mm, in compliance with the approved animal protocol.

### 4.9 Mouse tumor model for flow cytometry phenotyping

For two experiments, RAG2^−/−^, γc^−/−^ mice were engrafted with MDA-MB-231 cells by injecting the cells to the mammary fat pad and treated with vehicle or PTOX. BALB/c mice were engrafted with Ctrl^KO^ and CD47^KO^ 4T1 cells injected into the mammary fat pad and treated with vehicle or PTOX. The resultant tumors were collected, minced into pieces with diameters less than 1 mm, and dissociated in RPMI-1640 with 2 mg/mL Collagenase I (ThermoFisher), Hyaluronidase (MilliporeSigma), and DNase I (ThermoFisher) at 37 °C for 1 h to achieve a single-cell suspension. Cells were pelleted by centrifugation, and red blood cells were lysed with ACK lysis buffer, washed twice with FACS buffer (PBS with 2% FBS) and filtered through a 45 μm cell strainer. Samples were treated with FcR blocker (Miltenyi) before being stained with the indicated antibodies and subjected to flow cytometry analysis.

### 4.10 Flow cytometry analysis

Anti-mouse CD47 (clone miap301; BioLegend), anti-human CD47 (clone B6H12; BD Biosciences), anti-mouse F4/80 (clone BM8; BioLegend), anti-mouse CD11b (clone M1/70; BioLegend), anti-mouse MHC-II (clone M5/114.15.2; BioLegend), anti-mouse CD206 (clone C068C2; BioLegend), anti-mouse CD45 (clone 30-F11; BioLegend), and anti-mouse Gr-1 (clone RB6-8C5; BioLegend) were used for FACS analyses. Sytox blue was used to exclude dead cells. Annexin V (BD Biosciences) and 7-aminoactinomycin D (7-AAD; ThermoFisher) were used to evaluate cell viability of macrophages and MDA-MB-231 cancer cells with PTOX treatment. Flow cytometry analysis was performed on a BD LSRFortessa X-20 or Cytek Aurora, and cell sorting was conducted using a BD FACSAria III.

### 4.11 Bulk RNA sequencing

The MDA-MB-231 cells were pretreated with DMSO (Control) or 1 μM PTOX for 24 h, after which total RNA was extracted with an RNA extraction kit (Qiagen) and used for library preparation. A concentration of 1 μg of RNA was used for cDNA library construction and bulk RNA sequencing. The samples were submitted to Novogene for RNA sequencing. The libraries were sequenced on the Illumina NovaSeq 6000 platform. Raw sequencing reads were processed by removing adapter sequences, poly-N regions, and low-quality reads to generate clean reads. Next, mapped reads were obtained through aligning clean reads to the reference using Spliced Transcripts Alignment to a Reference (STAR V.2.7.8a). Differential gene expression analysis was performed using DESeq2, and results were expressed as log_2_ fold change (log_2_FC) with associated adjusted *p* value. Genes with an absolute fold change FC > 1.5 and adjusted *p* value < 0.01 were considered to be differentially expressed genes (DEGs). Gene set enrichment analysis (GSEA) and KEGG pathway enrichment were performed using the GSEA software, the Molecular Signatures Database, and visualized in RStudio.

### 4.12 ELISA

Tumor cells (Ctrl^KO^ and TNFα^KO^) were seeded in 24-well plates and allowed to adhere overnight. Cells were treated with PBS or 1 μM PTOX for 24 h. After treatment, culture supernatants were collected and removed the cell debris. The clarified conditioned media were subjected to TNFα quantification using a commercially available human TNFα ELISA kit (BioLegend) according to the manufacturer's instructions. Briefly, standards and samples were added to pre-coated ELISA plates and incubated for 2 h at room temperature. After washing, detection antibody and HRP-conjugated secondary reagent were sequentially added. Signal was developed using TMB substrate and stopped with stop solution. Absorbance was measured at 450 nm using a microplate reader. TNFα concentrations were calculated based on the standard curve. All measurements were performed with three independent biological replicates.

### 4.13 Statistical analysis and graphing software

Schematic illustrations were drawn with BioRender (Biorender.com). Flow cytometry data were analyzed using FlowJo V10.0 (TreeStar). The bulk of the data were statistically analyzed and graphed using Microsoft Excel or GraphPad Prism V.10. Mouse bioluminescence imaging (BLI) quantification and images were processed with Aura Imaging software (Spectral Instruments Imaging). Bulk RNA sequence data were processed with Partek Flow (Partek, an Illumina company) and visualized with RStudio (posit.co). Gene set enrichment analysis (GSEA) was performed with GSEA software (gsea-msigdb.org).

All data are presented as mean ± SD. Statistical significance between groups was determined using an unpaired *t*-test or one-way/two-way ANOVA. *p* value < 0.05 was considered statistically significant.

## CRediT authorship contribution statement

**Jessica Dang:** Writing – review & editing, Writing – original draft, Visualization, Validation, Software, Project administration, Methodology, Investigation, Formal analysis, Data curation, Conceptualization. **Nuozi Song:** Writing – original draft, Visualization, Validation, Software, Methodology, Formal analysis. **Jingkai Zhou:** Visualization, Validation, Methodology, Formal analysis, Data curation. **Mustafa Raoof:** Supervision. **Mingye Feng:** Writing – review & editing, Supervision, Project administration, Investigation, Funding acquisition, Methodology, Resources, Validation.

## Declaration of competing interest

The authors declare that they have no competing interests.
