# Supplementary material for: Podophyllotoxin sensitizes triple-negative breast cancer cells to CD47-targeted immunotherapy
Source: Cell Insight. 2026 Mar 13;5(3):100313. doi: 10.1016/j.cellin.2026.100313 (PMC13089164; doi:10.1016/j.cellin.2026.100313)
Supplement: Multimedia component 1 [file mmc1.docx]

**Supplemental Figures 1-3**

**Podophyllotoxin sensitizes triple-negative breast cancer cells to CD47-targeted immunotherapy**

Jessica Dang^1^, Nuozi Song^1^, Jingkai Zhou^1^, Mustafa Raoof^2,3^, Mingye Feng^1^

**Affiliations:**

^1^ Department of Immuno-Oncology, Beckman Research Institute, City of Hope, Duarte, CA, 91010, USA.

^2^ Department of Surgery, City of Hope, Duarte, CA, 91010, USA.

^3^ Department of Cancer Genetics and Epigenetics, Beckman Research Institute, City of Hope, Duarte, CA, 91010, USA.

^*^ Correspondence should be addressed to Mingye Feng, Ph.D., [mfeng@coh.org](mailto:mfeng@coh.org)

**Competing interests:** The authors declare that they have no competing interests.

**
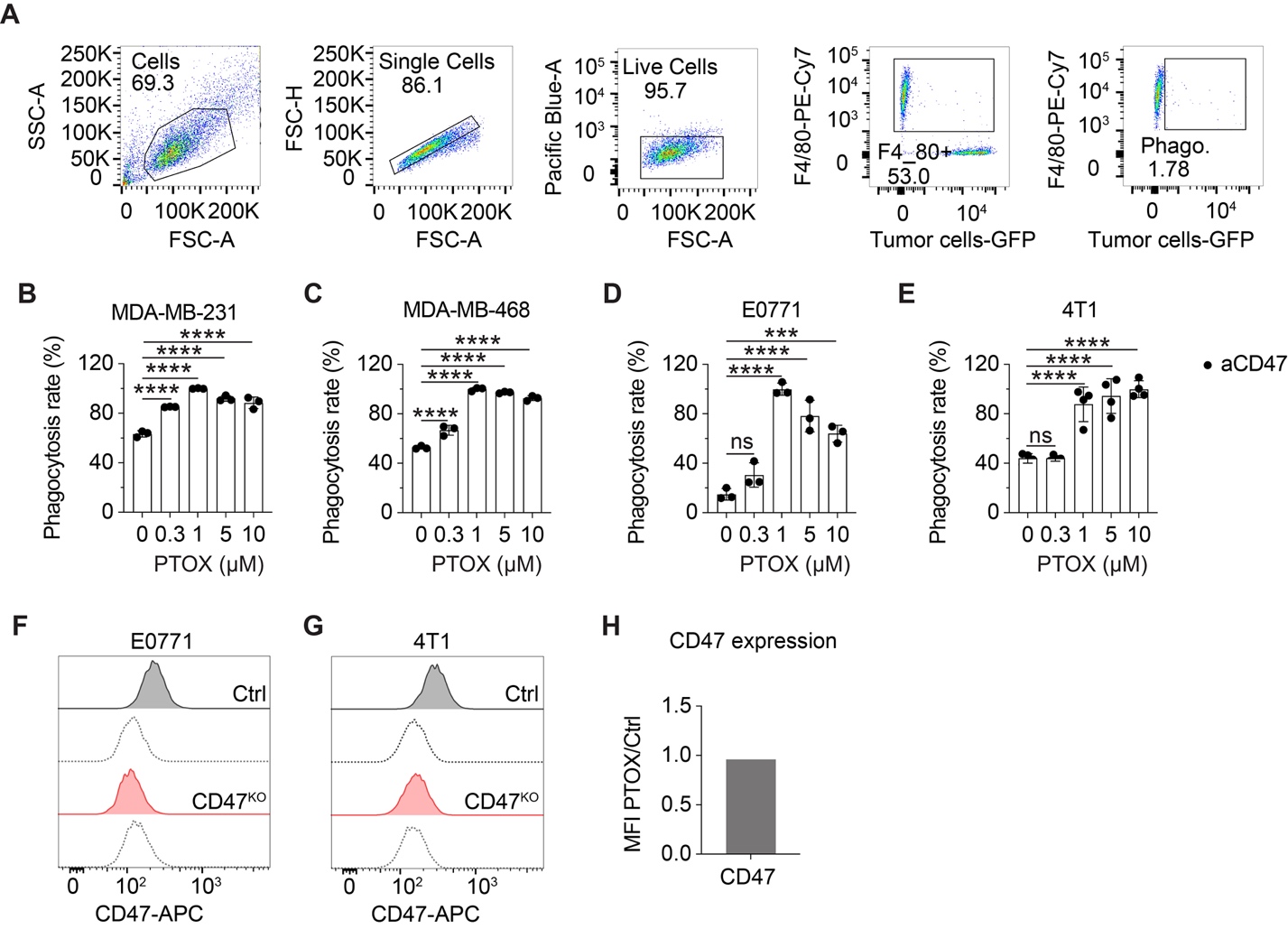
**

**Figure S1. The phagocytosis assay and expression of CD47 in TNBC cells.** (**A-E**) Flow cytometry-based phagocytosis assay of BMDMs and 4T1 cells co-cultured for 2 h in the presence of CD47-blocking antibody. TNBC cells with various concentrations of PTOX (0, 0.3, 1, 5, 10 μM). FACS gating strategy (A) and quantitative analysis (B-E). *n* = 3; two-way ANOVA with multiple comparison tests. **(F-G)** FACS plots showing the expression of CD47 in Ctrl^KO^ and CD47^KO^ E0771 (F) and 4T1 (G) cells. (**H**) MFI of CD47 expression after PTOX or PBS treatment in MDA-MB-231 cells. ns, no significance, *****P* < 0.0001. Error bars represent ± SD.


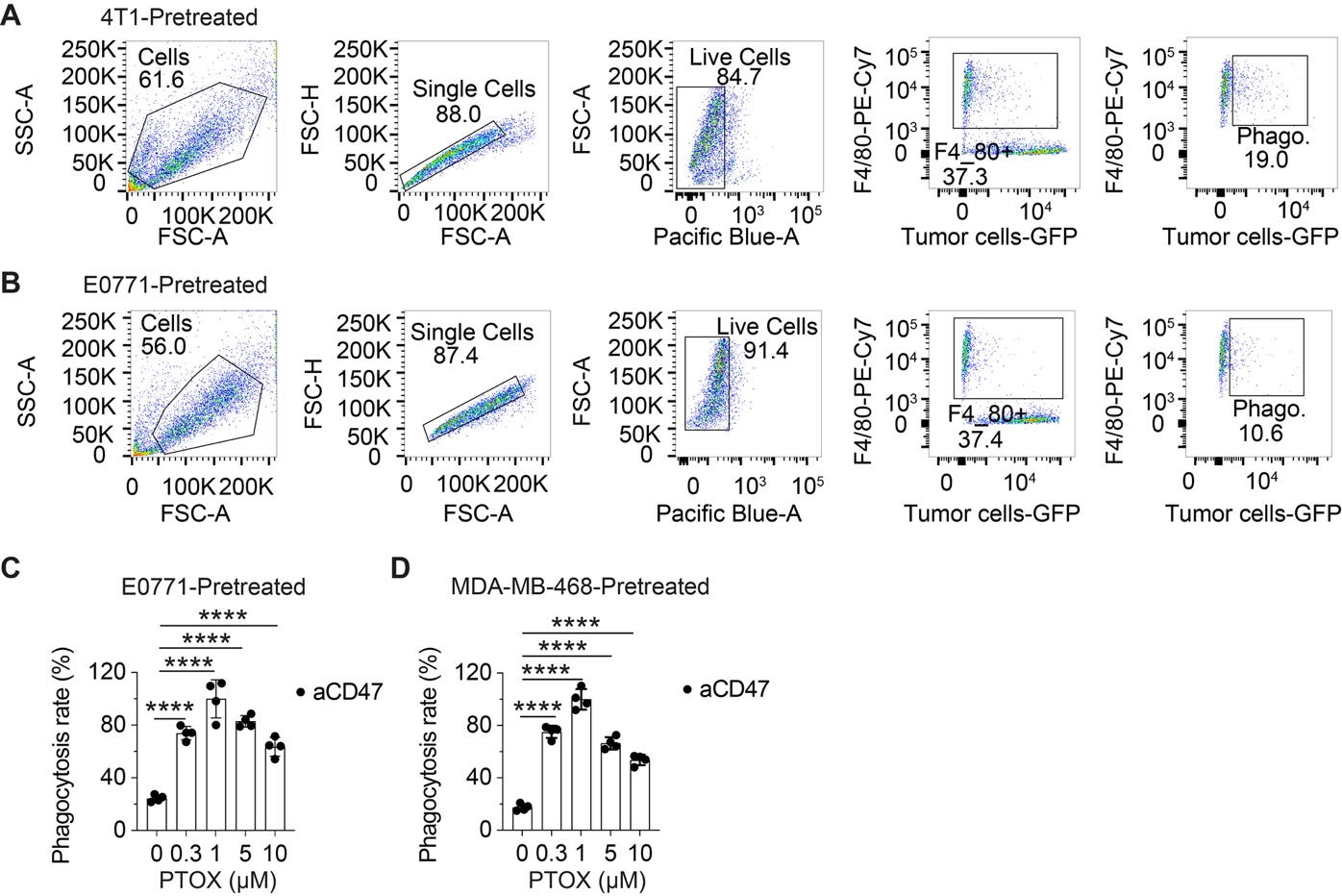


**Figure S2. The phagocytosis assay of 4T1, E0771, and MDA-MB-468 cells. (A)** FACS gating strategy of BMDMs and 4T1 cells which pretreated with PTOX co-cultured for 2 h in the presence of CD47-blocking antibody. **(B-C)** FACS gating strategy (B) and quantitative analysis (C) of phagocytosis in BMDMs and E0771 cells pretreated with PTOX and co-cultured for 2 h in the presence of CD47-blocking antibody. *n* = 4; two-way ANOVA with multiple comparison tests. **(D)** A luminescence-based phagocytosis assay with MDA-MB-468 cells as target cells. TNBC cells were pretreated with 1 μM of PTOX in the presence of an anti-CD47 blocking antibody. *n* = 4; one-way ANOVA with multiple comparison tests. *****P* < 0.0001. Error bars represent ± SD.


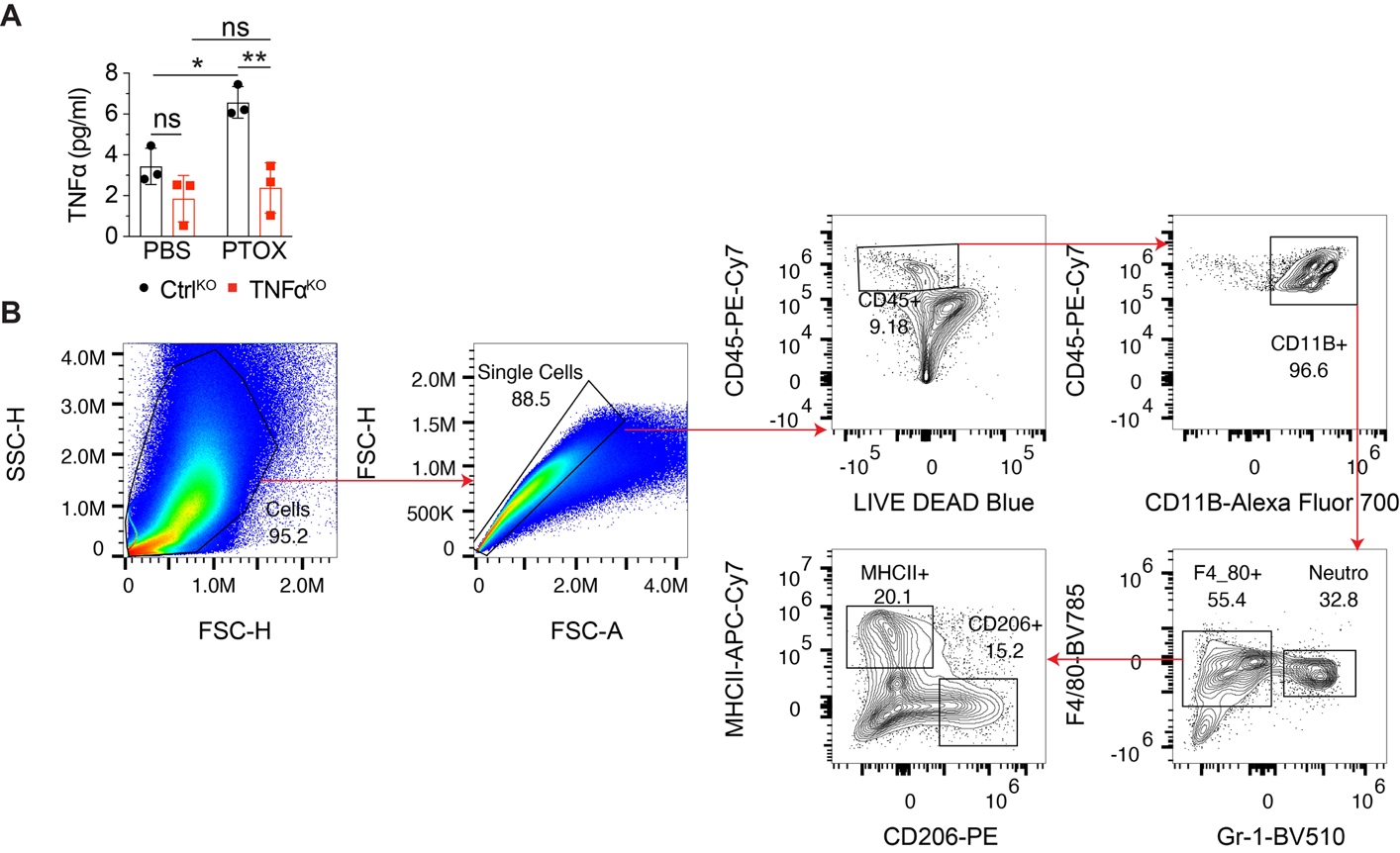


**Figure S3. PTOX alters immune cell composition in TNBC tumors.** (**A**) TNFα levels in conditioned media from Ctrl^KO^ and TNFα^KO^ TNBC cells treated with PBS or PTOX were measured by ELISA. (**B**) FACS gating strategy showing the percentage of immune (CD45+), CD11B+ myeloid cells, neutrophils, and F4/80+ TAM in tumor tissues from MDA-MB-231-engrafted mice treated with PTOX. ns, no significance, **P* < 0.05, ***P* < 0.01. Error bars represent ± SD.
