# Supplementary material for: Podophyllotoxin sensitizes triple-negative breast cancer cells to CD47-targeted immunotherapy
Source: Cell Insight. 2026 Mar 13;5(3):100313. doi: 10.1016/j.cellin.2026.100313 (PMC13089164; doi:10.1016/j.cellin.2026.100313)
Supplement: Multimedia component 2 [file mmc2.pdf]

Number	CAS	Compound Name	NSC	MW	UHAZ
1	66-22-8	2,4(1H,3H)-Pyrimidinedione	3970	112	
2	4005-51-0	1,3,4-Thiadiazol-2-amine	4728	101	
3	150-76-5	Phenol, 4-methoxy-	4960	124	
4	95-54-5	1,2-Benzenediamine	5354	108	TOX
5	543-21-5	2-Butynediamide	65381	112	
6	533-75-5	2,4,6-Cycloheptatrien-1-one, 2-hydroxy-	89303	122	
7	156-54-7	Sodium n-butyrate	174280	111	
8	23438-23-5	2,5-Cyclohexadien-1-one, 4-hydroxy-4-methyl-	295156	124	
9	622-78-6	Benzene, (isothiocyanatomethyl)-	118976	149	
10	41729-52-6	4H-Imidazo[4,5-c]pyridin-4-one, 6-amino-1,5-dihydro-	261726	150	
11	148-24-3	8-Hydroxyquinoline	285166	145	
12	-	3-Nitro-5-formylisoxazole	626433	142	
13	-	-	635968	141	
14	134-58-7	7H-1,2,3-Triazolo[4,5-d]pyrimidin-7-one, 5-amino-1,4-dihydro-	749	152	
15	50-44-2	6H-Purine-6-thione, 1,7-dihydro-	755	152	IRT TOX
16	499-44-5	2,4,6-Cycloheptatrien-1-one, 2-hydroxy-4-(1-methylethyl)-	18804	164	
17	672-76-4	2,4,6-Cycloheptatrien-1-one, 2-hydroxy-5-(1-methylethyl)-	18805	164	
18	20535-83-5	1H-Purin-2-amine, 6-methoxy-	37364	165	
19	55659-41-1	1-Pentanone, 1-(3-furanyl)-4-hydroxy-	349438	168.19	HTX
20	7467-33-6	5,6-Quinolinedione	400944	159	IVP
21	-	-	603719	151	
22	-	Furan, 2,5-dihydroxy-3,3,4,4-tetramethyl-	614928	160	
23	-	-	695218	159	
24	3565-26-2	8-Quinolinol, 5-nitroso-	3852	174	
25	58-27-5	1,4-Naphthalenedione, 2-methyl-	4170	172	
26	148-18-5	Sodium diethyldithiocarbamaate	4857	172	IRT TOX
27	483-55-6	1,4-Naphthalenedione, 2-hydroxy-3-methyl-	11897	188	
28	1780-33-2	4,6-dichloro-2,5-dimethylpyrimidine	40212	177.03	
29	4342-03-4	1H-Imidazole-4-carboxamide, 5-(3,3-dimethyl-1-triazenyl)-	45388	182.18	CRC IRT TOX
30	10108-64-2	Cadmium chloride	51148	183	CRC TOX
31	28989-50-6	-	54297	182	
32	5016-18-2	Pyrimido[5,4-e]-as-triazine-5,7(6H,8H)-dione, 6-methyl-	99733	179	
33	-	-	111041	189.17	
34	6478-73-5	-	326397	187	
35	79174-47-3	-	369317	180	
36	-	-	369318	190	
37	-	-	625639	178	
38	56-25-7	4,7-Epoxyisobenzofuran-1,3-dione, hexahydro-3a,7a-dimethyl-	61805	196	CRC
39	3073-59-4	N,N'-Hexamethylenebisacetamide	95580	200	
40	3814-79-7	Hydrazinecarbothioamide, 2-[(3-hydroxy-2-pyridinyl)methylene]-	95678	196	
41	2700-23-4	4-Nitrobenzylidenemalononitrile	98447	199	
42	37076-68-9	2,4(1H,3H)-Pyrimidinedione, 5-fluoro-1-(tetrahydro-2-furanyl)-, (R)-	148958	200	TOX
43	-	5-Isoxazoleacetic acid, .alpha.-amino-3-chloro-4,5-dihydro-4-hydroxy-	176324	195	
44	-	-	620050	198	
45	-	1,2,4,5-tetrazine, 3,6-bis(1-azetidinyl)-	625748	192	
46	-	-	664298	204	
47	-	-	673912	191	
48	-	4-[[(2-FURANYL)METHYL]AMINO]-1H-PYRAZOLO[3,4-D]PYRIMIDINE	1620	215.21	
49	484-11-7	1,10-Phenanthroline, 2,9-dimethyl-	4280	208	IRT
50	6374-92-1	5,7-Dichloroindole-2,3-dione	26045	216	IRT TOX
51	82499-04-5	1H-Purin-2-amime, 6-(methylsulfonyl)-	63446	213	
52	884-68-4	Securinan-11-one, (2.alpha.)-	107415	217	
53	18091-77-5	Pyrido[4',3':4,5]imidazo[1,2-c][1,2,3]benzotriazine	128734	221	
54	61895-38-3	5,8-Quinolinedione, 7-amino-6-methoxy-2-methyl-	132493	218	
55	28277-68-1	-	159935	224	
56	27978-30-9	Acetamide, 2-chloro-N-[4-(methylthio)phenyl]-	166464	216	
57	79127-35-8	1,5-(Epoxymethano)cyclopenta[c]pyran-3-carboxamide, 1,4a,5,7a-tetrahy	357683	223	
58	77-53-2	1H-3a,7-Methanoazulen-6-ol, octahydro-3,6,8,8-tetramethyl-, [3R-(3.alpha	403883	222	
59	-	-	606532	213	
60	-	-	635441	220	
61	-	-	635975	206	
62	-	-	657449	212	
63	-	-	666526	223	
64	-	-	688795	220	
65	-	-	693053	220	
66	137-26-8	Thioperoxydicarbonic diamide ([(H2N)C(S)]2S2), tetramethyl-	1771	240	IRT RPT TOX
67	1148-79-4	2,2':6',2"-Terpyridine	3905	233	HTX IRT IVP

68	3054-70-4	4-Pyrimidinol, 2,6-diamino-5-phenylazo-	5200	230	
69	117-10-2	9,10-Anthracenedione, 1,8-dihydroxy-	7210	240	CRC IRT
70	128-95-0	9,10-Anthracenedione, 1,4-diamino-	7833	238	CRC TOX
71	84-79-7	1,4-Naphthalenedione, 2-hydroxy-3-(3-methyl-2-butenyl)-	11905	242	
72	2507-55-3	Tetradecanoic acid, 2-hydroxy-	18938	244	
73	50-89-5	2,4(1H,3H)-Pyrimidinedione, 1-(2-deoxy-.beta.-D-erythro-pentofuranosyl)-	21548	242	
74	6265-55-0	-	33004	227	
75	6622-22-6	-	56544	228	
76	58-81-1	2,4,6-trichloro-5-propylpyrimidine	66914	226	
77	4238-67-9	-	86100	234	
78	4238-66-8	6H-Pyrido[4,3-b]carbazole, 5-methyl-	87206	232	
79	53878-12-9	-	99027	239	
80	39935-49-4	2,4(1H,3H)-Pyridinedione, 1-.beta.-D-ribofuranosyl-	126849	243	
81	-	Hydrazinecarbothioamide, N-methyl-2-[(5-nitro-2-thienyl)methylene]-	169543	244	
82	-	4(1H)-Quinazolinone, 2,3-dihydro-2-(5-methyl-2-thienyl)-	175634	244	
83	-	-	299879	235	
84	71555-25-4	[1-(2-pyridinyl)ethylidene] hydrazide	319726	234.32	IVP
85	30990-64-8	6H-[1,2,5]Oxaselenazolo[4,3,2-hi][2,1,3]benzoxaselenazole-3-SeIV, 7,8-dihy	328587	245	
86	107089-76-9	4H-1,2,4-Benzothiadiazine, 3-chloro-4-methyl-, 1,1-dioxide	373853	231	
87	1811-23-0	(5aS,9aS)-6,6,9a-trimethyl-4,5,5a,6,7,8,9,9a-octahydronaphtho[2-c]furan-	375294	234	
88	90597-22-1	2(1H)-Pyrimidinone, 4-amino-1-[4,5-dihydroxy- 3-(hydroxymethyl)-2-cyclo	375575	239	IVP PSN
89	4955-79-7	-	379531	231	
90	-	Benzene, 1-[[2-chloro-1-(chloromethyl)ethyl]thio]-4-fluoro-	607347	239	
91	-	1-(2'-Deoxy-2'-fluoro-D-arabinofuranosyl)- 1,2-pyrimidine-2-one	625355	230	
92	-	-	635563	238	
93	-	Tris(acetylthio)methane	637833	238	
94	-	1,4-Benzenediol, 2,5-bis(1-pyrazolyl)-	645987	242	
95	-	-	664286	241	
96	-	-	664331	228	
97	-	4-(benzothiazol-2-yl)-2-methyl-benzenamine	674495	240	IRT
98		2,5-Diaziridinyl-3-(hydroxymethyl)- 6-methyl-1,4-benzoquinone	697726	234	ALK
99	518-75-2	3H-2-Benzopyran-7-carboxylic acid, 4,6-dihydro-8-hydroxy-3,4,5-trimethy	186	250	CRC IVP RPT TOX
100	55-98-1	1,4-Dimethanesulfonyloxybutane	750	246.29	CRC HTX IVP
101	98-77-1	1-Piperidinecarbodithioic acid,	1906	246	
102	5327-10-6	Morpholinium-N-oxydiethylenedithiocarbamate	4114	250	
103	81-54-9	9,10-Anthracenedione, 1,2,4-trihydroxy-	10447	256	IRT RPT
104	6259-19-4	3,7-Dibenzothiophenediamine, 5,5-dioxide	13966	246	IVP
105	2529-64-8	3-Deoxyestradiol	22194	256	
106	-	-	29603	263	
107	7059-23-6	Hydrazinecarboximidamide, 2,2'-(1-methyl-1,2-ethanediylidene)bis-, dihyd	32946	257.13	
108	6951-16-2	-	54650	253	
109	73-03-0	3'-Deoxyadenosine	63984	251	IRT RPT TOX
110	550-33-4	9-(beta-D-Ribofuranosyl)-9H-purine	65423	252.23	
111	443-30-1	N,N-Dimethyl-.alpha.-indolylidene-p-toluidine	80087	247	
112	6754-13-8	(3aR,5R,5aR,8aR,9S,9aS)-9-hydroxy-5,8a-dimethyl-1-methylidene-3a,4,5,5	85236	262.3	TOX
113	18883-66-4	2-Deoxy-2-(3-methyl-3-nitrosoureido)-D-glucopyranose	85998	265.22	CRC RPT
114	14173-81-0	6-amino-7-bromo-5,8-dihydroquinoline-5,8-dione	105808	253	
115	6756-41-8	-	106296	262	
116	70857-52-2	1-(chloromethyl)naphthalene	145150	260	
117	27089-56-1	2-[3,4-dihydroxy-5-(hydroxymethyl)oxolan-2-yl]-3-sulfanylidene-1,2,4-tr	146268	261	IVP
118	316-46-1	5-Fluorouridine	146604	262	IVP
119	844-63-3	3-(2-hydrazono-2-phenylethylidene)-1,3-dihydro-2H-Indol-2-one	175274	263	
120	3009-42-	2-[2-(1,3-benzodioxol-5-yl)ethenyl]-, (Z)-benzoxazole	176655	265	
121	-	-	191384	265	
122	19358-23-7	N-(2,4-dimethylphenyl)-3-phenylacrylamide	191389	251	
123	75968-21-7	[[(4-methoxyphenyl)sulfonyl]hydrazono]-acetic acid	267213	258	
124	-	L-Arabinose, 2-amino-4-[(2-amino-1-oxopropyl)amino]-2,4-dideoxy-, monc	278619	256	
125	64124-21-6	[1,3,5-triazine-2,4,6-triyltris(methylimino)]tris-methanol	283162	258.28	
126	60084-10-8	2-beta-D-ribofuranosylthiazole-4-carboxamide	286193	260.26	
127	62584-08-1	12-Hydroxyindolo[2,1-b]quinazoline-6(12H)-one	292147	250	
128	70015-86-0	N-(2-chloroethyl)-N-nitroso-N'-(3-pyridinylmethyl)-, N-oxide	307454	259	ALK
129	76235-60-4	1-Naphthalenecarboxylic acid, 5,6,7,8-tetrahydro-2-hydroxy-4-methyl-7-(1	310618	246	
130	67199-66-0	[1,2-b]quinazolin-12(10H)-one, 8-amino-Isoindolo	320846	249.27	
131	97534-21-9	5-Pyrimidinecarboxamide, hexahydro-4,6-dioxo-N-phenyl-2-thioxo-	336628	263	IRT
132	27108-13-2	2-Propen-1-one, 1-(2-chlorophenyl)-2-[(dimethylamino) methyl]-, hydrochl	382007	260	
133	1146-04-9	Spiro[cyclopropane-1,5'-[5H]inden]-7'(6'H)-one, 2',3'-dihydro-3',6'-dihydro	400978	248	IVP TOX
134	958-11-2	2,5-Cyclohexadiene-.delta.1,.alpha.-acetonitrile, .alpha.-(p-chlorophenyl)-	405158	257	
135	-	-	634471	265	

136	-	-	634650	252	
137	-	Naphthalene-1,2-dione, 1,2-dihydro-4-[(3-pyridinyl)amino]-	637729	250	
138	-	Phenoxybenzylidene-malononitrile	643028	246	
139	-	Phenoxybenzylidene-malononitrile	643031	246	
140	-	-	663996	246	
141	-	-	667235	248	
142	-	2-Propenenitrile, 3-[3-(dimethylamino)phenyl]-2-phenyl-	667251	248	
143	-	-	676561	247	
144	7761-45-7	2,4-Pyrimidinediamine, 5-(3,4-dichlorophenyl)-6-methyl-	7364	269	IVP
145	112-80-1	9,10-Octadecenoic acid	9856	282	
146	10453-89-1	2-(1-Isobutenyl)-3,3-dimethylcyclopropanecarboxylic acid	11779	168	
147	81424-67-1	N-[(methylamino)carbonyl]-N-[[(methylamino)carbonyl]oxy]-acetamide	253272	189	
148	35832-09-8	1,3,5-Triazine-2,4,6-triamine, N,N,N',N',N''-pentamethyl-, monohydrochlor	118742	233	
149	-	Benzo[g]quinoxaline-5,10-dione, 5,10-dihydro-2,3-dimethyl-	602617	238	
150	52-52-8	Cyclopentanecarboxylic acid, 1-amino-	1026	129	HTX
151	5854-93-3	L-Alanine, 3-(hydroxynitrosoamino)-	529469	149	
152	51-18-3	1,3,5-Triazine, 2,4,6-tris(1-aziridinyl)-	9706	204	ALK CRC
153	20537-88-6	2-(3-Aminopropylamino)ethyl thiophosphate	296961	214.22	TOX
154	83730-53-4	2-amino-4-(S-butylsulfonimidoyl)-butanoic acid	326231	222.3	RPT
155	21339-68-4	1-benzothieno[2,3-g]isoquinoline, 5,11-dimethyl-	119686	263	
156	42228-92-2	(2S)-2-amino-2-[(5S)-3-chloro-4,5-dihydro-1,2-oxazol-5-yl]acetic acid	163501	179	TOX
157	10102-18-8	Disodium selenite	347466	175	HTX
158	13494-90-1	Nitric acid, gallium salt	15200	259	IRT OXI
159	1910-42-5	4,4'-Bipyridinium, 1,1'-dimethyl-, dichloride	263500	257	TOX
160	-	Naphtho[2,3-d]thiazole-4,9-dione, 3-methyl-2-(methylimino)-	659501	258	
161	135-49-9	3,6-Acridinediamine, 2,7-dimethyl-, monohydrochloride	13973	274	IRT RPT TOX
162	118-00-3	2-Amino-1,9-dihydro-9-.beta.-D-ribofuranosyl-6H-purin-6-one	19994	283	TOX
163	479-13-0	6H-Benzofuro[3,2-c][1]benzopyran-6-one, 3,9-dihydroxy-	22842	268.23	IRT IVP RPT TOX
164	6306-63-4	1-(2-carboxyphenyl)-3-(dimethyl-amino)-2-methylpropan-1-one	22992	271.74	
165	6265-57-2	-	33006	268	
166	92025-69-9	1H-Purin-2-amine, 6-[(2-phenylethyl)thio]-	35866	271	
167	6296-08-8	3,5,7-Triaza-1-azoniaadamantane (8CI), 1-(2-thienyl)-, chloride	49660	273	
168	69-74-9	2(1H)-Pyrimidinone, 4-amino-1-.beta.-D-arabinofuranosyl-, monohydrochl	63878	280	IRT IVP RPT
169	10371-86-5	6H-Pyrido[4,3-b]carbazole, 9-methoxy-5,11-dimethyl-	69187	276	
170	3228-71-5	9H-Purin-6-amine, 9-.alpha.-D-arabinofuranosyladenine	70422	267	
171	2133-81-5	6H-Purine-6-thione, 2-amino-9-(2-deoxy-.alpha.-D-erythro-pentofuranosyl)	71851	283	TOX
172	10299-44-2	3H-1,2,3-Triazolo[4,5-d]pyrimidin-7-amine, 3-.beta.-D-ribofuranosyl-	72961	268	
173	-	-	74420	273	
174	525-58-6	2-methyl-3-[(2-methyl-3H-indol-3-ylidene)methyl]-indole	76747	272	
175	392-29-0	1,3-Butanedione, 1-benzo[b]thien-3-yl-4,4,4-trifluoro-	80396	272	
176	2507-91-7	3-Ethoxy-2-oxobutyraldehyde bis(thiosemicarbazone)	82116	276	
177	60132-23-2	4H-Cyclopent[f]oxacyclotridecin-4-one, 1,6,7,8,9,11a.beta.,12,13,14,14a.	89671	280.36	IVP TOX
178	56282-16-7	-	97703	274	
179	6742-12-7	1H-Pyrazolo[4,3-d]pyrimidine, 7-amino-3-.beta.-D-ribofuranosyl-	102811	267	
180	4291-63-8	2-chloro-2'-deoxy-adenosine	105014	285.69	IRT RPT TOX
181	36417-16-0	1,4-Naphthalenedione, 2-(3,3-dichloro-2-propenyl)-3-hydroxy-	126771	283.11	TOX
182	21416-87-5	2,6-Piperazinedione, 4,4'-propylenedi-	129943	268.27	TOX
183	31191-21-6	-	139109	282	
184	31785-60-1	4(1H)-Quinazolinone, 2,3-dihydro-2-(1-naphthyl)-	145669	274	IVP
185	78695-17-7	(5-bromo-1H-benzimidazol-2-yl)-, methyl ester	154754	270	
186	-	-	155595	284	
187	40448-85-9	1(2H)-Quinolinecarbothioic acid, 2-cyano-6-methoxy-, S-ethyl ester	165897	274	IVP
188	24613-06-7	2,6-Piperazinedione, 4,4'-(1-methyl-1,2-ethanediyl)bis-, (R)-	169779	268	ALK
189	12128-65-3	carbonyl(.eta.(5)-2,4-cyclopentadien-1-yl)(dimethylcarbamodithioato-S,S')	175493	269	
190	-	5-Hydroxy-2-pyridinecarboxaldehyde, 4-(3-pyridinyl)thiosemicarbazone	185065	273	
191	73108-81-3	-	191392	272	
192	-	-	191393	272	
193	33205-72-0	-	208914	280	
194	62402-31-7	5,6-Dihydro-5-azacytidine hydrochloride	264880	283	
195	2270-41-9	Trichothec-9-ene-3,4,15-triol, 12,13-epoxy-, (3.alpha.,4.beta.)-	269142	282	ALK
196	10403-51-7	4,8-Ethenopyrrolo[3',4':3,4]cyclobut[1,2-f]isoindole-1,3,5,7(2H,6H)tetron	284356	272	
197	69408-81-7	1H-Benz[de]isoquinoline-1,3(2H)-dione, 5-amino-2-[2-(dimethylamino)ethy	308847	283	IRT IVP RPT TOX
198	5536-17-4	9H-Purin-6-amine, 9.beta.-D-arabinofuranosyl-	404241	267	
199	892-49-9	9-.beta.-D-Arabinofuranosyl-1,9-dihydro-6H-purine-6-thione	406021	284	
200	518-82-1	4,5,7-Trihydroxy-2-methylanthraquinone	408120	270	
201	-	-	605756	268	
202	-	Cyclopentanemethanaminium, N,N,N-trimethyl-2-oxo-, iodide	621889	283	
203	-	2-(chloromethyl)-1,3-dinitro-5-(trifluoromethyl)benzene	622627	284.57	

204	-	-	622640	279	
205	-	-	622684	275	
206	-	-	625641	277	
207	-	1-[(4-methylphenyl)sulfonyl]-2-nitro-benzene	627708	277	
208	-	-	632233	285	
209	-	-	635404	281	
210	-	-	636817	283	
211	-	1,2-dione, 1,2-dihydro- 4-[(2-phenylethyl)amino]-naphthalene	637731	277	
212	-	N-[6-[(1H-pyrrol-1-yl)methyl]- 1-benzimidazol-2-yl]-, ethyl ester	639754	284	
213	-	-	640624	272	
214	-	2-(3,4-Dimethoxybenzylidene)-1-indanone	643174	280	
215	-	2-Naphthylidene benzoylacetonitrile	643186	283	
216	-	-	643910	276	
217	140674-76-6	Benzoic acid, 4-[[(2,5-dihydroxyphenyl)methyl]amino]-, methyl ester	654705	273	
218	150258-65-4	-	655255	274	
219	-	2-(1-Piperazino)naphthazarin	658144	274	
220	-	-	687667	278	IRT
221	-	2-(2-(1-(pyrimidin-4-yl)ethylidene)hydrazinyl)benzo[d]thiazole	693632	269.32	
222	-	2-Nitro-5-p-toluenesulfonylfuran	697923	267	
223	6639-96-9	-	12825	300	
224	6364-25-6	2(10H)-Phenazinone, 8-amino-10-phenyl-	31702	287	
225	5069-77-2	Purine, 2-amino-6-[(o-chlorobenzyl)thio]-	36826	292	
226	2653-64-7	2-Naphthalenol, 1-(1-naphthalenylazo)-	45575	298	
227	606-58-6	7-Deaza-7-cyanoadenosine	63701	291	IVP
228	33581-08-7	2',4'-Dimethyl-3-nitrosalicylanilide	79456	286	
229	1603-46-9	-	95848	300	
230	15663-27-1	Platinum, diamminedichloro-, (SP-4-2)-	119875	300.06	CRC HTX IRT IVP
231	-	5H-Pyrido[3,4-b][1,4]benzothiazine-5-ethanol, acetate (ester)	168221	286	
232	25528-10-3	-	175296	286	
233	-	Uracil, 5-[2-[1-(hydroxymethyl)-3-methylbutyl]amino]- carbonyl]ethenyl]6-	224117	295	
234	61827-19-8	-	233872	299	
235	4682-50-2	Trichothec-9-en-4-ol, 12,13-epoxy-, acetate, (4.beta.)-	267033	292	ALK
236	66929-50-8	Urea, N-[2-[(2-chloroethyl)thio]ethyl]-N'-cyclohexyl-N-nitroso-	292684	294	ALK
237	19622-83-4	Margetine lycoricidine	349155	291	
238	528-48-3	5-Desoxyquercetin	407010	286	
239	-	-	407335	288	
240	-	Imidazole, 1-(4-chlorophenyl)-4-(4-nitrophenyl)-	610744	300	
241	-	-	611750	289	
242	-	-	622616	292	
243	-	1H,3H-Thiazolo[3,4-a]benzimidazole, 1-(2,6-difluorophenyl)-	625487	288	IVP
244	-	Tetracyanoethylene-Cyclohepta[cd]benzofuran Diels Alder Adduct	629301	296	
245	-	-	630374	295	
246	-	-	631583	299	
247	-	-	634863	292	
248	-	-	636786	290	
249	-	2-(3,4-Dimethoxy benzylidene)-1-tetralone	643175	294	
250	-	-	643774	290	
251	-	Naphth[2,3-d]oxazol-9-one, 2-methyl-4-(phenylimino)-	650573	288	
252	-	Thiophene-2-methanol, 5,5'-(2,5-furandiyl)bis-	652287	292.37	
253	-	2-Hydroxy-N-(4-methoxyphenyl)-4-oxo-4-phenylbut-2-enamide	658709	297.31	
254	-	Naphthalene-1,4-dione, 2-chloro-5,8-dihydroxy- 3-(2-methoxyethoxy)-	659997	299	
255	106148-11-2	Pyrazolo[3,4-d][1]benzazepin-5-one, 1,4,5,6-tetrahydro-3-methyl-1-phenyl	662553	289	
256	-	-	680516	290	
257	514-10-3	Podocarpa-7,13-dien-15-oic acid, 13-isopropyl-	25149	302	
258	475-91-2	1,3-Dioxolo[4,5-b]acridin-10(5H)-one, 4,11-dimethoxy-5-methyl-	34757	313	
259	53-19-0	2,4'-Dichlorodiphenyldichloroethane	38721	320.05	CRC IVP
260	53584-29-5	CHIMYL ALCOHOL	59269	317	
261	18417-89-5	7-Deazaadenosine-7-carboxamide	65346	309	HTX IVP
262	-	Guanidine, 1-(p-butoxyphenyl)-3-(p-chlorophenyl)-	78365	317.82	
263	63981-15-7	2-Hydroxy-N-(2-methyl-5-nitrophenyl)-3-nitrobenzamide	79451	317	
264	73454-90-7	2',3-Dinitro-p-salicylotoluidide	82025	317	
265	14077-73-7	-	92510	310	
266	2126-70-7	2-Butenoic acid, 3-bromo-4-(4-methoxyphenyl)-4-oxo-, sodium salt, (Z)-	104801	308	
267	26786-84-5	1-Phenazinecarboxylic acid, 6-formyl-4,7,9-trihydroxy-, methyl ester	106995	314.25	
268	4712-12-3	5,7-Dihydroxy-3',4'-dimethoxyflavone	128305	314	
269	22862-75-5	3,4-Pyrrolidinediol, 2-(p-methoxybenzyl)-, 3-acetate, hydrochloride, cis-2,3	147340	302	IVP
270	35943-35-2	1,4,5,6,8-Pentaazaacenaphthylene, 3-amino-1,5-dihydro-5-methyl-1-.beta	154020	320	IVP
271	-	5,6-Dimethyl-2-[(3,3,3-trifluoro-2-trifluoromethyl)propyl]benzoxazole	168415	309	

272	34934-08-2	4(1H)-Quinazolinone, 6-chloro-2,3-dihydro-2-(1-naphthalenyl)-	175636	309	IVP
273	-	-	202000	308	
274	33396-49-5	CC 9095	208913	308	
275	29477-83-6	[1,2]Dioxolo[4,5-j]phenanthridin-6(2H)-one, 3,4,4a,5-tetrahydro-2,3,4,7-t	266535	307.26	RPT
276	73544-88-4	3'-Chloro-3-nitro-o-salicylotoluidide	328477	307	
277	-	-	329279	308	
278	82585-91-9	Carbamic acid, (5-amino-1,2-dihydro-3-phenylpyrido[3,4-b]pyrazin-7-yl)-, e	330770	311	IVP
279	77691-03-3	9-Deazaadenosine	352890	302.72	IVP
280	-	Dibromodulcitol	616232	308	
281	13243-65-7	1,4-Naphthalenedione, 2,3-diphenyl-	618332	316	
282	83665-54-7	1H-Pyrido[3,4-b]indole, 2,3,4,9-tetrahydro- 1-[3-(trimethylsilyl)-3-penteny	619165	313	
283	-	4-N,N-bis-2'-cyanoethylaminobenzylidene-4-fluoro aniline	620277	320	
284	-	-	622732	309	
285	-	1H,3H-Thiazolo[3,4-a]benzimidazole, 1-(2-chloro-6- fluorophenyl)-	625483	305	
286	-	-	625590	315	
287	-	3(2H)-Pyridazinone, 2-(2-benzothiazolyl)-6-phenyl	629659	307	
288	-	-	631152	303	
289	-	Pyridine, 3-[4-methyl-5-[(1-methyl-4-nitro-1H-imidazol-5-yl)thio]- 4H-1,2,4	631160	317	
290	-	Benzene, 1,1'-sulfonylbis(2-nitro-	633001	308	
291	-	-	637914	314	
292	-	-	664327	307	
293	-	1,4-Thiazino[3,2-c]quinoline-3-thione, 2-phenyl-	667467	308	
294	-	-	680506	301	
295	-	-	682864	310	
296	1578-05-8	1,1,3-Tribromo-3-methyl-2-butanone	1027	322.82	
297	2058-71-1	-	4810	327	
298	5453-77-0	-	18891	334	
299	465-92-9	8.beta.H-Labda-13(16),14-dien-19-oic acid, 15,16-epoxy-6.beta.,9-dihydro	36693	332.44	
300	82-89-3	2,2'-Bi-1H-pyrrole, 4-methoxy-5-[(5-methyl-4-pentyl-2H-pyrrol-2-ylidene)m	47147	323.44	IVP
301	-	-	65937	322	
302	2878-62-8	4-(2,4,5-Trimethoxystyryl)quinoline	80756	321	
303	632-99-5	4-Amino-m-tolyl-bis(4-aminophenyl)methane chloride	93739	338	CRC
304	5544-25-2	1H-Pyrrolo[2,1-c][1,4]benzodiazepine-2-acrylamide, 5,10,11,11a-tetrahydr	106408	329	HTX IVP
305	21231-30-1	-	118735	331	
306	-	Cyclohexanone, 2,6-bis(piperidinomethyl)-	39202	292	
307	-	-	636126	295	
308	-	-	636132	292	
309	2121-16-6	Lycobetaine chloride	338259	302	
310	-	Naphtho[2,1-b]quinolizinium, 7-methyl-, chloride	28002	280	
311	-	Cyclopentanone, 2,5-bis[(dimethylamino)methyl]-, cis-, dihydrochloride	640391	271	
312	-	-	666168	290	
313	-	-	68093	318	PSN
314	21231-35-6	-	118732	310	
315	-	1-Indenone, 3-hydroxy-2-(2-quinoxalinyl)-	634224	274	
316	-	-	41809	286	
317	-	-	635140	297	
318	60342-56-5	L-Aspartic acid, N-(phosphonoacetyl)-, disodium salt	224131	301	
319	-	1H-Pyrrolo[1,2-a]benzimidazole-5,8-dione, 6-(1-aziridinyl)- 2,3-dihydro-3-(	651079	315	ALK IVP
320	26801-94-5	Acenaphtho[1,2-b]quinoxaline, 7,12-dioxide	60309	286	
321	40939-76-2	Isothiocyanic acid, sulfonyldi-m-phenylene ester	140911	332	
322	10048-13-2	7H-Furo[3',2':4,5]furo[2,3-c]xanthen-7-one, 3a,12c-dihydro-8-hydroxy-6-m	204985	324	CRC TOX
323	57999-04-9	-	224124	336	
324	64894-81-1	Benzoic acid, 4-(9-acridinylamino)-, monosodium salt	235082	337	
325	22551-45-7	Eunicin	282752	334	
326	21887-01-4	HORMINON	294577	332	
327	70452-30-1	3-Phenacyliden-5-brom-2-indolinon	294961	328	
328	69895-67-6	9,10-Anthracenedione, 1-hydroxy-4-[[2-[(2-hydroxyethyl)amino]ethyl]amir	299187	326.35	
329	51123-99-0	2,4-Quinazolinediamine, 6-[[3-(trifluoromethyl)phenyl]thio]-	305782	336	
330	14846-62-9	4-Nitroestrone 3-methyl ether	321803	329	
331	85619-28-9	6H-Pyrido[4,3-b]carbazole-1-carboxamide, 5,11-dimethyl-, monohydrochl	335142	326	
332	108030-77-9	Pyridine, 3,5-dichloro-2,4-dimethoxy-6-(trichloromethyl)-	338720	325	IVP
333	97919-22-7	Benzenesulfonamide, 4-amino-N-[5-chloro-2-quinoxalinyl]-	339004	335	
334	96203-70-2	[1,3]Dioxolo[4,5-j]phenanthridin-6(2H)-one, 1,3,4,4a,5,11b-hexahydro-1,2	349156	325	IVP
335	-	-	360861	332	
336	25176-29-8	2,4-Imidazolidinedione, 3,3'-(1,6-hexanediyl)bis[5,5-dimethyl-	376791	338	
337	-	-	603108	336	
338	-	-	621094	325	
339	-	1,2,4-Dithiazol-3-amine, 5-[(2-furanyl)methylimino]- N,N-dimethyl-, monol	622608	322.24	

340	-	-	622690	340	
341	-	-	623135	332	
342	-	Benzoic acid, [(6-methyl-2-oxo-2H-benzopyran- 4-yl)methoxy]-, methyl este	623637	324	
343	-	-	624161	326	
344	-	Tricyclo[4.4.0.0(2,7)]decane-5-one, 4,4-dibromo-2-methyl-	624358	322	
345	-	Piperidine, 4-(p-anilinoanilino)-2,2,6,6-tetramethyl-	632536	323.48	
346	-	4-Piperidinone, 3,5-bis[(4-methylphenyl)methylene]-, hydrochloride	632839	340	
347	-	4-Piperidinone, 1-(1-oxo-2-propenyl)-3,5-bis(phenyl- methylene)-	632841	329	
348	-	-	634658	324	
349	-	-	635121	325	
350	-	-	635306	334	ALK
351	118112-10-0	N-[3-(2-Pyridyl)isoquinolin-1-yl]-2-pyridinecarboxamidine	637578	325	IVP
352	-	3-Bromo-4-dimethylamino benzalacetophenone	643164	330	
353	-	-	645617	329	
354	-	-	648422	328	
355	-	6-N-aziridinyl-7-methyl-2,3-dihydro-1H-pyrrolo[1,2-a]benzimidazole-5,8-d	651080	336	ALK IVP
356	-	-	658285	338	
357	-	1H-Pyrido[3,4-b]indol-1-one, 2-[4-(dimethylamino)butyl]-2,3,4,9-tetrahyc	658388	336	
358	-	-	664329	336	
359	313-67-7	Phenanthro[3,4-d]-1,3-dioxole-5-carboxylic acid, 8-methoxy-6-nitro-	11926	341	CRC RPT TOX
360	-	-	20514	343	
361	2150-48-3	Ammonium, (6-diethylamino-3H-xanthen-3-ylidene)diethyl-, chloride	44690	359	
362	1168-42-9	4',5,6,7-Tetramethoxyflavone	53908	342	
363	466-49-9	Aspidospermidine, 1-acetyl-17-methoxy-	61811	354	
364	595-05-1	(4bS,5R,10bS,11R)-5,6,11,12-Tetrahydro-13,18-dimethyl-5,10b:11,4b-bis	99016	346	
365	5058-45-7	Neo-Oxyberberine	123390	352	
366	-	-	135996	353	
367	21090-35-7	7H-Pyrrolo[2,3-d]pyrimidine-5-carboxamide, 4-amino-7-.beta.-D-ribofurar	143648	346	HTX IVP
368	22417-22-7	Sulfonium, tri-p-tolyl-, chloride	157930	341	
369	-	Pentanamide, 2-(acetylamino)-N-[3-chloro-2-oxo-1-(phenylmethyl)propyl]-	173905	353	
370	22897-08-1	7H-Furo[3',2':4,5]furo[2,3-c]xanthen-7-one, 3a,12c-dihydro-8-hydroxy-6,1	178249	354	
371	-	2-Propenamide, N-[2-(butylsulfinyl)-1-(hydroxymethyl)ethyl]-3-(1,2,3,4-tet	241509	357	
372	23593-75-1	1-(o-Chlorophenyldiphenylmethyl)imidazole	257473	345	IRT TOX
373	15529-90-5	Gold, chloro(triethylphosphine)-	313981	351	IRT TOX
374	-	9,10-Anthracenedione, 1,4-dihydroxy-2-[[2-[(2-hydroxyethyl)amino]ethyl]a	316157	342	
375	-	-	320864	350	
376	79514-43-5	3-Azabicyclo[3.2.2]nonane-3-carboselenoic acid, [1-(2-pyridinyl)ethylidene	323241	349.34	HTX IVP
377	-	Piperazine-1-carbothioic acid, 4-cycloheptyl-, [1-(2-pyridyl)ethylidene]hydr	376265	359.53	IVP
378	-	-	382766	354	
379	-	-	383468	350	
380	-	-	616355	350	
381	-	Methyl 13-hydroxy-15-oxo-kaurenoate	620358	346	
382	-	-	631529	341	
383	-	Imidazo[2,1-b]thiazole-5-carboxamide, N-[(4-chlorophenyl)-aminocarbony	633209	355	
384	-	-	634232	344	
385	-	2H-1,4-Benzothiazin-3(4H)-one, 2-[2-[(3-chloro-2-methylphenyl)amino]-2-c	634396	347	
386	-	-	637680	342	
387	-	-	642649	359	
388	-	3-Bromo-4-dimethylamino benzylidene-1-tetralone	643162	356	
389	-	2-(3-Bromo-4-dimethylamino benzylidene)-1-indanone	643163	342	
390	-	-	646189	350	
391	-	-	646200	346	
392	-	-	647613	358	
393	-	1H-Pyrrolo[1,2-a]benzimidazole-5,8-dione, 6-(acetylamino)- 2,3-dihydro-3-	651084	347	
394	-	-	671424	358	
395	-	Hexadecanaminium, N-(chloromethyl)-N,N-dimethyl-, chloride	672904	354	
396	124341-23-7	1H-Benzimidazole-4-carboxamide, N-[2-(dimethylamino)ethyl]-2-(4-pyridir	678932	346	
397	-	Propanoic acid, 3,3,3-trifluoro- 2-[(4-fluorophenyl)amino]-2-[[(ethoxy)carb	684845	352	
398	5335-97-7	4-[[(8-hydroxyquinolin-7-yl)-phenylmethyl]amino]benzoic acid	1011	370.41	
399	548-57-2	1-[[2-(Diethylamino)ethyl]amino]-4-methylthiaxanthone hydrochloride	14574	377	TOX
400	5459-31-4	-	24113	369	
401	6165-03-3	6-Benzylthiopurine ribonucleoside	26273	374	
402	458-37-7	1,6-Heptadiene-3,5-dione, 1,7-bis(4-hydroxy-3-methoxyphenyl)-	32982	368.39	IRT
403	28028-68-4	5-Hydroxy-2,3-dihydrophthalazine-1,4-dione	36437	376	
404	2799-07-7	S-Triphenylmethyl-L-cysteine	83265	363.47	TOX
405	-	Hydrazinecarboximidamide, 2-[1-[4-(4-acetylphenoxy)phenyl]ethylidene]-,	97911	373	
406	5852-02-8	-	115538	366	
407	16910-79-5	Azirino[2',3':3,4]pyrrolo[1,2-a]indole-4,7-dione, 1,1a,2,8,8a,8b-hexahydro	123115	376.41	

408	23444-70-4	2-Butenoic acid, 3-methyl-, 1-(5,8-dihydro-1,4-dihydroxy-5,8-dioxo-2-nap	140377	370	
409	56401-88-8	Pyridine, 2-(p-chlorostyryl)-4-[[4-(diethylamino)-1-methylbutyl]amino]-, (E	157389	372	
410	633-65-8	Berbinium, 7,8,13,13a-tetradehydro-9.10-dimethoxy-2,3-(methylene diox	163088	372	
411	39966-41-1	Mercury, chloro-2-naphthalenyl-	174163	363	TOX
412	57998-68-2	1,4-Cyclohexadiene-1,4-dicarbamic acid, 2,5-bis(1-aziridinyl)-3,6-dioxo-, d	182986	364	ALK TOX
413	51984-29-3	Acetic acid, (2-chlorophenoxy)-, 6-[[(aminothioxomethyl)hydrazono]methy	185056	365	
414	24338-53-2	Podolactone B, 7,8-deepoxy-8,14-didehydro-15,16-dideoxy-7-hydroxy-, (7	211500	362	
415	58449-06-2	1-Propanone, 1-[2,4-dihydroxy-3-[(2-hydroxyphenyl)methyl]-6-methoxyph	241906	378	
416	61848-62-2	1,2-Cyclohexanediamine, platinum complex, (1S-trans)-	265459	380	
417	73211-11-7	sodium bis(2-ethylhexyl) sulfosuccinate	302979	374	
418	-	-	327697	361	
419	38077-12-2	1-Butanone, 1-(4-fluorophenyl)-4-[4-[(4-fluorophenyl)hydroxymethyl]-1-pi	343513	373	
420	-	Ethanol, 2-[[6-[1-(3,4,5-trimethoxyphenyl)ethyl]-1,3-benzodioxol-5-yl]oxy]	352876	376	
421	143-62-4	3.beta.,14-Dihydroxy-5.beta.-card-20(22)-enolide	407806	374.52	HTX
422	-	-	601101	373	
423	-	-	604535	369	
424	-	Phenol, 4,4'-(2-pyridinylmethylene)bis-, diacetate (ester)	614826	361.4	IRT TOX
425	-	-	617540	361	
426	-	-	620279	374	
427	-	4-Piperidinone, 2,6-bis(4-methoxyphenyl)-3,5-dimethyl-1-nitroso-	626734	368	
428	-	-	629713	362	
429	-	1H-Pyrano[3',4':6,7]indolizino[1,2-b]quinoline-3,14(4H,12H)-dione, 10-an	629971	363	IVP
430	-	Naphtho[2,3-d]thiazole-4,9-dione, 2-[(3,4-dichlorophenyl) amino]-	631521	375	
431	-	-	635437	371	
432	-	Copper, bromo[2-[1-(2-pyridinyl)ethylidene][N,N-dimethyl-hydrazinecarbo	635448	365	IVP
433	-	-	635542	370	
434	-	-	640580	375	
435	-	Pyrimidine, 2-[1,5-bis(4-chlorophenyl)-1H-1,2,3-triazol-4-yl]-1,4,5,6-tetral	645033	372	
436	-	-	647363	372	
437	-	2,2'-(1,3-phenylenebis(methylene))bis(azanediyl)bis(2-phenylacetonitrile)	681730	366.46	
438	-	-	681741	368	
439	5394-71-8	4-hydroxy-3-[3-(4-phenoxyphenyl)propyl]naphthalene-1,2-dione	377	384.43	
440	64-86-8	Acetamide, N-(5,6,7,9-tetrahydro-1,2,3,10-tetramethoxy-9-oxobenzo[a]he	757	399	HTX IRT IVP RPT
441	1448-22-2	Glaucarubol	14974	396	
442	568-53-6	Naphtho[2,3-d]-1,3-dioxole-6-carboxylic acid, 5,6,7,8-tetrahydro-9-hydrox	24817	400	IVP TOX
443	806-29-1	6.alpha.,9.alpha.-Difluoroprednisolone	77021	396	
444	38714-92-0	Acetophenone, 2'-[(6,7-dimethoxy-1-isoquinolyl)methyl]-4',5'-dimethoxy-	98542	381	IRT TOX
445	18776-75-5	2-Oxazolin-5-one, 4-[p-[bis(2-chloroethyl)amino] benzylidene]-2-phenyl-	104117	389	ALK
446	-	DESMETHOXY-B-PELTATIN-A METHYL ETHER	126727	398	IVP
447	-	Antibiotic AT 116 benzylamine salt	136037	383	
448	-	9H-Thioxanthen-9-one, 4-(hydroxymethyl)-1-[[2-(4-methyl-1-piperazinyl)et	166381	384	
449	-	1-Phenanthrenecarboxylic acid, 10-bromo-1,2,3,4,4a,9,10,10a-octahydro	169600	395	
450	7336-33-6	Acetamide, N-(5,6,7,9-tetrahydro-3-hydroxy-1,2,10-trimethoxy-9-oxobenz	172946	385	HTX IVP
451	51264-14-3	Methanesulfonamide, N-[4-(9-acridinylamino)-3-methoxyphenyl]-	249992	393	CRC IVP TOX
452	32884-24-5	-	262665	385	
453	1239-45-8	2,7-Diamino-10-ethyl-9-phenylphenanthridinium bromide	268986	394	
454	84173-29-5	COMPACTIN	281245	391	
455	24372-73-4	Piperidinium, 1-[4-(4-methoxyphenyl)-1,3-dithiol-2-ylidene]-, sulfate	302358	390	
456	55435-65-9	Acetamide, N-methyl-N-[4-[(7-methyl-1H-imidazo[4,5-f]quinolin-9-yl)amino	305884	382	
457	-	-	322069	396	
458	19186-35-7	Furo[3',4':6,7]naphtho[2,3-d]-1,3-dioxol-6(5aH)-one, 5,8,8a,9-tetrahydro-!	403148	398	IVP TOX
459	-	Platinum, dichloro[dimethylsilylenebis(methanamine)]-, (SP-4-2)	603577	384	
460	41941-56-4	Adenosine, 8-chloro-, cyclic 3',5'-(hydrogen phosphate)	284751	364	
461	81721-80-4	-	337612	337	
462	-	-	635337	337	
463	-	-	636084	335	
464	-	-	600305	352	
465	-	2H-1-Benzopyran-2-one, 4-[(tetrahydro-4-methylene-5-oxo-2-phenyl-2-fur	668270	348	
466	63520-86-5	-	293927	338	
467	306-67-2	1,4-Butanediamino, N,N'-bis(3-aminopropyl), tetrahydrochloride	69852	348	IRT
468	-	Acridine, 2-methoxy-6-nitro-9-(2-phenylhydrazino)-	689872	360	
469	66358-49-4	5H-[1,3]Dioxolo[5,6]indeno[1,2-c]isoquinoline-5,12-dione, 2,3-dimethoxy	314622	365	
470	-	-	329277	378	
471	-	Naphtho[2',3'-4,5]thiazole[2,3-g]purine-2,4,7,12-tetrone, 1,3-dimethyl-	634568	366	
472	13063-04-2	[1,3]Benzodioxolo[5,6-c]phenanthridinium, 2,3-dimethoxy-12-methyl-, ch	146397	384	
473	-	-	371846	398	
474	-	2-Propen-1-one, 1-(1,4-dioxo-2,6,7-trimethylquinoxalin-3-yl)-3-(2-nitroph	621486	379	
475	-	Dibenzofuran-1,3(2H,9bH)-dione, 2,6-diacetyl-7,9-dihydroxy-8,9b-dimeth	5890	344	

476	-	1-Piperidineethanol, .alpha.-[p-(p-chlorostyryl)phenyl]-	26040	341.88	
477	-	-	163443	394.47	
478	-	-	623051	383	
479	35982-88-8	2H,4H-Oxazolo[5,4,3-ij]pyrido[3,2-g]quinoline-4,10(11H)-dione, 8-[(acetyl	157004	340	
480	-	Ergosterol	62791	397	
481	-	-	624169	393	
482	-	-	634473	381	
483	-	-	635321	392	
484	-	5,7-dichloro-3-hydroxy-3-[2-(4-nitrophenyl)-2-oxoethyl]-1,3-dihydro-2H-ir	635326	381.17	
485	-	-	635328	386	
486	-	5-chloro-3-hydroxy-7-methyl-3-(2-oxo-1,2-diphenylethyl)-1,3-dihydro-2H-	635435	391.85	
487	-	-	635438	381	
488	-	-	644735	395	
489	-	-	657456	399	
490	-	1-Phenazinecarboxamide, N-[2-(dimethylamino)ethyl]- 6,9-dimethoxy-, mc	678917	391	
491	-	-	680509	388	
492	-	7H-Pyrido[3,2-d][1]benzazepin-6-one, 2-(3-chlorophenyl)- 5,6-dihydro-4-pl	684480	397	
493	207350-09-2	-	705330	385	
494	-	ACETOXYPHENANTHRYLMERCURY	534	437	TOX
495	59-05-2	L-Glutamic acid, N-[4-[[2,4-diamino-6-pteridinyl)methyl]- methylamino]be	740	454.44	ALK IRT IVP RPT TOX
496	125-20-2	1(3H)-Isobenzofuranone, 3,3-bis[4-hydroxy-2-methyl-5-(1-methylethyl)phe	2186	431	
497	-	7,8,8A,9-TETRACHLOROTETRADECAHYDRO-7-ISOPROPYL-1.BETA.,4A.BETA.-	2979	444	
498	6329-82-4	-	11930	418	
499	69-05-6	3-Chloro-7-methoxy-9-[1-methyl-4-(diethylamino)butylamino]acridine dih	14229	472.88	IRT
500	67-98-1	1-[4-(2-Diethylaminoethoxy)phenyl]-1-phenyl-2-(p-anisyl)ethanol	19857	420	
501	5459-47-2	-	24048	442	
502	518-28-5	Naphtho[2,3-d]-1,3-dioxole-6-carboxylic acid, 5,6,7,8-tetrahydro-8-hydrox	24818	414	HTX IRT IVP
503	518-29-6	Furo[3',4':6,7]naphtho[2,3-d]-1,3-dioxol-6(5aH)-one, 5,8,8a,9-tetrahydro-	24819	414	IVP TOX
504	63989-75-3	Benzamide, N-(5,6,7,9-tetrahydro-1,2,3,10-tetramethoxy-9-oxobenzo[a]he	33410	462	IVP TOX
505	7401-24-3	-	35489	475	
506	-	-	35949	470	
507	-	-	39863	417	
508	13153-25-8	9H-Purine, 2-amino-6-[(o-chlorobenzyl)thio]-9-.beta.-D-ribofuranosyl-	40666	423.87	
509	155-58-8	.beta.-D-Glucopyranoside, 3-hydroxy-5-[2-(3-hydroxy-4-methoxyphenyl)et	43321	420	
510	3801-06-7	Pregna-1,4-diene-3,20-dione, 17-(acetyloxy)-9-fluoro-11-hydroxy-6-methyl	47438	419	IVP
511	6625-20-3	2-Naphthacenecarboxamide, 4-dimethylamino-1,4,4a,5,5a,6,11,12a-octal	51812	451	
512	-	Propanoic acid, 2,2'-thiobis-, bis[[(3-methoxyphenyl)methylene]hydrazide]	56737	443	
513	-	Benzenesulfonic acid, 4-hydroxy-2-methyl-5-(1-methylethyl)-, compd. with	66300	415	
514	6965-36-2	-	67580	432	
515	6954-59-2	-	67690	405	
516	70460-30-9	-	71300	402	
517	-	-	71669	412	
518	3618-58-4	1-Naphthalenesulfonic acid, 3-hydroxy-4-[(2-hydroxy-1-naphthalenyl)azo]-	73413	462.39	
519	3607-17-8	Phosphonium, (3-bromopropyl)triphenyl- bromide	84074	464	
520	518-88-7	3-hydroxy-4-((1-hydroxynaphthalen-2-yl)diazenyl)-7-nitronaphthalene-1-su	85561	462.39	
521	6759-89-3	Quinolinium, 2-[2-(8-hydroxy-5-quinolyl)vinyl]-1,6-methyl-, iodide	85700	454.31	
522	545-47-1	Lup-20(29)-en-3-ol, (3.beta.)-	90487	427	
523	13896-99-6	Thiazolo[3,2-f]phenanthridinium, 3-phenyl- perchlorate	98904	412	
524	14937-58-7	-	100856	409	
525	15986-25-1	s-Triazine, 4,6-diamino-1-[p-[4-(2,4-dichlorophenyl)butyl]phenyl]-1,2-dihy	104129	455	
526	-	Lipoxamycin, sulfate (salt)	106997	422	
527	14509-96-7	Lobinaline, monohydrochloride	109444	423	
528	472-15-1	Lup-20(29)-en-28-oic acid, 3.beta.-hydroxy-	113090	457	
529	34409-15-9	2(1H)-Pyrimidinone, 4-amino-1-[2,3,5-tris-O-(1-oxobutyl)-.beta.-D-arabino	138429	453	
530	23255-93-8	Thioxanthen-9-one, 1-[[2-(diethylamino)ethyl]amino]-4-(hydroxymethyl)-,	142982	453	
531	63-45-6	1,4-Pentanediamine, N4-(6-methoxy-8-quinolinyl)-, phosphate (1:2)	149765	455	
532	-	4H-1-Benzopyran-4-one, 3-[(deoxyhexosyl)oxy]-2-(3,4-dihydroxyphenyl)-6,	167410	448	
533	13302-14-2	Plumbane, tributylchloro-	168597	414	IVP
534	2062-78-4	1-[1-[4,4-Bis(p-fluorophenyl)butyl]-4-piperidyl]-2-benzimidazolinone	170984	461.54	
535	-	Carbamic acid, [1-[[[3-chloro-2-oxo-1-(phenylmethyl)propyl]amino]carbon	173904	445	
536	56457-41-1	Mercury, (acetyloxy)(pentamethylphenyl)-	174176	407	TOX
537	21288-60-8	e-Rhodomycinone	196524	428	
538	-	-	211489	456	
539	62928-11-4	Platinum, dichlorodihydroxybis(2-propanamine)-, (oc-6-33)-	256927	418	
540	61786-74-1	2-Propenamide, N-[2-(decylthio)-1-(hydroxymethyl)ethyl]-3-(1,2,3,4-tetrah	265473	426	
541	62523-06-2	Carbamic acid, ethyl-, [5-(3,4-dichlorophenyl)-2,3-dihydro-1H-pyrrolizine-	267700	454	
542	-	RESIBUFOGENIN, METHACRYLATE DERIV	267712	453	ALK
543	75919-74-3	N,N'-Bis(2-methyl-7-amino-4-quinolinyl)-1,7-heptanediamine	273829	443	

544	66922-25-6	Butanoic acid, 2-methyl-, 7-(acetyloxy)-2,3,3a,4,7,8,9,11a-octahydro-9-hy	292663	422	
545	87626-57-1	4H-1-Benzopyran-8-acetic acid, 4-oxo-2-phenyl-, 2-(diethylamino)ethyl este	293015	416	
546	81531-60-4	9-Hydroxy-2-(2-piperidinylethyl)ellipticinium acetate	311153	433.55	
547	-	HM-9-AO	329696	418	
548	69363-14-0	SCHISANHENOL	330515	402	
549	81086-04-6	2-Anthracenecarboxamide, N-[4-(diethylamino)-1-methylbutyl]-9,10-dihyc	331757	429	
550	71439-68-4	9,10-Anthracenedicarboxaldehyde, bis(4,5-dihydro-1H-imidazol-2-ylhydra	337766	471	IVP PSN
551	88181-19-5	-	345081	405	ALK
552	82423-05-0	4,6-Methano-5H-benz[h]oxazolo[3,2-a]pyrazino[3,2,1-de][1,5]naphthyridir	349644	426	IVP TOX
553	-	-	351306	435	
554	523-67-1	Magon	374898	411	TOX
555	-	-	600300	472	
556	-	Platinum, tetrachloro[dimethylsilylenebis(methanamine)]-, (OC-6-22)	603578	455	
557	-	2H-1-Benzopyran, 6-methoxy-3-nitro-2-[2,2,2',2'-tetramethyl (4,4'-bi-1,3-d	618261	407	
558	-	1H-Pyrazole-3-one, 4-[[[4-[bis(2-cyanoethyl)amino]phenyl] methylene]amir	620280	412	
559	-	-	623746	449	
560	111238-61-0	Benzenethiosulfonic acid, 4-methyl-, 2-butene-1,4-diyl ester, (Z)-	624158	429	
561	-	Pyrimidine-5-carboxamide, N-(4-chlorophenyl)-4- [2-[(4-chlorophenyl)metl	624947	416	
562	-	Methyl 3,5-di-O-(4-chlorobenzyl)-.alpha.-D-ribofuranoside	626120	413	
563	-	1H-Benzo[de]-1,3-benzodioxolo[5,6]quinoline-6a- carbonitrile, 6-benzoyl-1	627666	454	
564	-	1-Naphthalenecarboxamide, N,N'-1,8-(octanediyl)bis-	629738	453	
565	-	Acetamide, .alpha.-(2-chloro-1,4-dihydro- 1,4-dioxonaphthalen-3-yl)-.alph	634503	407	
566	-	-	634926	432	
567	-	-	634928	468	
568	-	5,7-dichloro-3-hydroxy-3-(2-oxo-1,2-diphenylethyl)-1,3-dihydro-2H-indol-	635312	412.27	
569	-	-	635436	405	
570	-	-	635824	420	
571	-	-	635833	412	
572	-	-	637916	450	
573	134742-26-0	Benzamide, 2-amino-N-[[[4-[(5-bromo-2-pyrimidinyl)oxy]- 3-chlorophenyl]a	639828	463	IVP
574	-	-	640974	442	
575	-	4-(2-fluorophenyl)-2,4-dioxo-3-(3-oxo-1,3-dihydroisobenzofuran-1-yl)-N-o-	641228	431.41	
576	-	-	641253	473	
577	-	-	641607	403	
578	-	-	642048	432	
579	-	-	643599	434	
580	-	-	647418	456	
581	-	-	648419	406	
582	-	1,4,7,10-Tetrathia-13-azacyclopentadecane, 13-[(4-methylphenyl)sulfonyl]	650792	438	
583	-	2-Propen-1-one, 1-[1-ethyl-4-hydroxy- 4-[2-(4-methylphenyl)ethenyl]-3-pip	657298	426	
584	-	Thiazolo[2,3-b]thiazolium, 2,3-dihydro- 2-[(2-methoxyphenyl)methylene]-!	657446	433	
585	-	Thiazolo[2,3-b]thiazolium, 2,3-dihydro- 2-[(4-nitrophenyl)methylene]- 5-(3	657598	448	
586	-	-	657603	446	
587	-	-	658293	413	
588	-	-	658494	450	
589	-	-	659174	423	
590	58086-84-3	6-Bromo-2,3,7-trichloro-3-(bromomethyl)-7-methyl-1-octene,(mixture of (	662825	401	
591	-	-	664181	416	
592	-	2-(5-Bromouracil-1-yl)-4-methylene-5-oxo-2-(p-phenylphenyl)-2,3,4,5-tetrl	668260	453	
593	-	2-((1-(2,4-Dichlorophenylmethyl)4-tert.butylcyclohexyl)oxy)N,N-dimethyl-	670224	423	
594	-	cis-2((1-(4-methyl-phenyl)methyl)-4-tert.butylcyclohexyl)oxyN,N-dimethyl	670225	448	
595	-	trans-2((1-(4-fluorphenyl-methyl)-4-tert.butylcyclohexyl)oxy)N,N-dimethyl	670226	452	
596	-	Ethanamine, N,N-dimethyl-2-[4-(1,1-dimethylethyl)- 1-[(4-methylphenyl)m	670229	448	
597	-	2-Pyrrolidinecarboxlic acid, 1-[.alpha.-(4-methoxyphenyl)-.alpha.- (3,4,5-t	673622	429	
598	-	Cyclopentanone, 3-[3-[(2-methoxyethoxy)methoxy]oct-1-enyl]- 4-[[dimethy	689228	429	
599	-	Benzoic acid, 4-[[(2,5-dihydroxyphenyl)methyl]amino]-, 1-adamantanemetl	689857	408	
600	-	-	693172	414	
601	-	-	697443	426	
602	-	-	698031	459	
603	466-06-8	14-Hydroxy-3.beta.-(rhamnosyloxy)bufa-4,20,22-trienolide	7521	530.66	HTX IVP
604	508-77-0	5.beta.-Card-20(22)-enolide, 3.beta.-(.beta.-D-cymarosyloxy)-5,14-dihydro	7522	549	
605	1448-23-3	Picras-3-en-16-one, 11,20-epoxy-1,2,11,12-tetrahydroxy-15-(2-hydroxy-2-	14975	497	
606	-	-	18298	572	
607	-	Benzo[a]heptalen-9(5H)-one, 7-acetamido-1-(glucosyloxy)-6,7-dihydro-2,3,	32992	548	
608	4727-50-8	1,1'-Diethyl-4,4'-carbocyanine iodide	34391	480	HTX
609	49720-72-1	Benzo[a]heptalen-9(5H)-one, 7-amino-6,7-dihydro-1,2,3,10-tetramethoxy-	36354	507	IVP TOX
610	3930-19-6	5-Amino-6-(7-amino-5,8-dihydro-6-methoxy-5,8-dioxo-2-quinolyl)-4-(2-hy	45383	506.47	HTX IVP RPT
611	6199-67-3	19-Norlanosta-5,23-diene-3,11,22-trione, 25-(acetyloxy)-2,16,20-trihydro	49451	558.71	IVP

612	303-45-7	8-Formyl-1,6,7-trihydroxy-5-isopropyl-3-methyl-2,2'-bisnaphthalene	56817	519	RPT
613	14415-43-1	-	79688	476	ALK
614	11033-34-4	1,6,11(2H)-Naphthacenetrione, 4-[(6-deoxy-2-O-methylhexopyranosyl)oxy]	93419	575	
615	3065-79-0	Benzothiazolium, 3-ethyl-2-[3-(3-ethyl-2(3H)-benzothiazolylidene)-2-methy	96932	506	
616	-	7-O-Methylnogalarol	102815	599.59	
617	17154-54-0	-	118030	482	
618	473-98-3	Lup-20(30)-ene-3.beta.,28-diol	4644	443	IRT
619	-	-	635366	405	
620	-	4-Piperidinone, 3,5-bis(phenylmethylene)-1-(1-oxo-3-phenyl- 2-propenyl)-	638634	406	
621	-	5H-Pyrido[3,2-d][2]benzazepin-6(7H)-one, 4-(3,4-dimethoxyphenyl)-2-pher	682769	422	
622	6312-47-6	1,4-Naphthalenedione, 2,2'-(1,4-piperazinediyl)bis[3-chloro-	40341	467	
623	10113-31-2	Tribenzyltin acetate	164909	451	TOX
624	-	1,3,2-Thiazastannolidine, 2,2-dibutyl-3-[(4-methylphenyl)sulfonyl]-	183359	462	
625	65956-63-0	Ethanaminium, 2-[[hydroxy(octadecyloxy)phosphinyl]oxy]-N,N,N-trimethy	282880	436	IVP
626	35290-22-3	Nordracorubin	376248	475	
627	-	-	622586	414	
628	-	-	622589	428	
629	-	-	624206	411	
630	-	6H-Imidazo[4,5,1-de]acridin-6-one, 5-[2-(diethylamino) ethylamino]-8-met	637993	451	
631	-	-	643148	425	
632	-	p-Dimethylaminocinnamic acid, copper complex	643351	444	
633	6286-09-5	N,N-Bis(2-methyl-6-methoxy-4-quinaldyl)-1,9-diamino- nonane Dihydrochl	10010	573.6	
634	-	3,5-Dioxa-4-phosphaunacosan-1-aminium, 4-hydroxy-N,N,N-trimethyl-, hy	605583	408	
635	-	-	623059	389	
636	-	1,2-Benzo-8-(L-alanyl)-3-phenoxazone nitrate salt	672425	397	
637	-	-	15623	464	
638	4660-54-2	-	48151	424	
639	-	-	90829	409	
640	25546-65-0	D-Streptamine, O-2,6-diamino-2,6-dideoxy-.alpha.-D-glucopyranosyl-(1.fw	138925	454	
641	3148-09-2	Spiro(16,18-methano-1H,3H,23H-[1,6,12]trioxacyclooctadecino[3,4-d][1]	126728	503	ALK HTX IRT IVP
642	14358-43-1	Emetine, 2,3-didehydro-, dihydrochloride, (+-)-	129414	552	TOX
643	1259-86-5	Picras-3-ene-2,16-dione, 11,20-epoxy-1,11,12-trihydroxy-15-(2-hydroxy-2	132791	495	
644	-	1-[3-chloro-4-(3-phenoxypropoxy)phenyl]-1,6-dihydro-6,6-dimethyl-1,3,5-	133071	512.02	
645	41191-04-2	[2-chloro-4-(4,6-diamino-2,2-dimethyl-s-triazin-1(2H)-yl)-phenoxy]-N,N-dir	139105	539	IVP TOX
646	73927-98-7	Tributyl(2,4,5-trichlorophenoxy)tin	164914	487	TOX
647	41451-75-6	2H-3,11c-(Epoxymethano)phenanthro[10,1-bc]pyran, picras-3-en-21-oic ac	165563	548.59	HTX IVP
648	3577-89-7	L-Leucine, N-[N-acetyl-4-[bis(2-chloroethyl)amino]-DL-phenylalanyl]-, ethyl	167780	488	ALK TOX
649	66421-94-1	Ethanol, 2-[[2-[4-[3-[2-(trifluoromethyl]-10H-phenothiazin-10-yl]propyl]-1-	169676	590	
650	14907-98-3	BRUSATOL	172924	521	
651	-	L-Glutamic acid, N-[[4-[[(2,4-diamino-6-pteridinyl)methyl]methylamino]-1-	174121	505	IVP
652	63345-17-5	1-Propanesulfonamide, 3-[(aminoiminomethyl)amino]-N-[4-[(3-nitro-9-acri	177365	566.46	
653	54965-24-1	Ethanamine, 2-[4-(1,2-diphenyl-1-butenyl)phenoxy]-N,N-dimethyl-, (Z)-, 2-h	180973	563.65	CRC IRT IVP RPT TOX
654	-	Trigilletine	181486	563	
655	26055-63-0	Aquayamycin	184398	486	
656	-	-	184403	575	
657	39669-49-3	Benzoic acid, 2-[[4-[bis(2-bromopropyl)amino]-2-methylphenyl]azo]-	240419	497	ALK
658	-	Ethanesulfonamide, N-[4-(9-acridinylamino)-3-methoxyphenyl]-, monomet	243928	504	
659	42936-80-1	Urea, N-(2-chloroethyl)-N-nitroso-N'-(2,3,4,6-tetra-O-acetyl-.beta.-D-glucop	245432	482	ALK
660	64202-81-9	17-Amino-17-demethoxygeldanamycin	255109	546	ALK IVP
661	70095-84-0	N,N-Dimethyldaunorubicin hydrochloride	258812	592	IVP PSN
662	668-17-7	1-Naphthacenecarboxylic acid, 2-ethyl-1,2,3,4,6,11-hexahydro-2,5,7,10-te	267229	586	IVP TOX
663	67642-36-8	.beta.-L-Glucopyranosylamine, 4,6-dideoxy-4-[[[(14-methyl-1-oxopentadec	268251	575.75	IVP
664	71628-96-1	7-O-Methylnogarol	269148	542	IVP
665	-	Verrucarin A, 7'-deoxo-2'-deoxy-4',8-dihydroxy-7'-(1-hydroxyethyl)-	269754	549	IRT IVP TOX
666	-	5H-Pyrrolo[2,1-c][1,4]benzodiazepin-5-one, 1,2,3,11a-tetrahydro-3,8-dihy	285223	525	
667	56457-10-4	1H-Benz[g]indazole-3-carboxamide,1-(4-chlorophenyl)- N-[2-(diethylamino	288010	489	
668	43000-65-3	17a-Aza-D-homoandrostan-17-one, 3-[[[4-[bis(2-chloroethyl)amino]phenyl]	290205	564	ALK
669	-	1H-Cycloundec[d]isoindol-1-one, 15-(acetyloxy)-2,3,3a,4,5,6,6a,9,10,11,1	305222	494	HTX RPT
670	-	1-(2,8 -is(trifluoromethyl)quinolin-4-yl)-3-(tert-butylamino)propan-1-ol, pl	305819	541.33	
671	87532-30-7	8B-Hydroxy-9B,10B-epoxyverrucarin A	328166	535	ALK IVP TOX
672	73341-73-8	Geldanamycin, 18,21-didehydro-6,17-didemethoxy-18,21-dideoxo-18,21-	330500	560.69	
673	-	Benzo[3,4]cycloocta[1,2-f][1,3]benzodioxole-5,6-diol, 5,6,7,8-tetrahydro-1	330516	537	
674	27548-93-2	Baccatin III	330753	587	
675	-	[9,9'-bi-4H-Naphtho[2,3-b]pyran]-4,4'-dione, 2,2',3,3'-tetrahydro-5,5',6,6',	345647	546.53	
676	-	6H-Benzo[d]naphtho[1,2-b]pyran-6-one,4-(6-deoxy-3-C-methyl-.beta.-D-gu	354844	508.52	IVP
677	-	4,8-Ethenopyrrolo[3',4':3,4]cyclobut[1,2-f]isoindole- 1,3,5,7(2H,6H)-tetroı	361813	497	
678	-	-	363744	513	
679	-	Anthracene-9,10-dione, 1,5-bis[3-[[(2-hydroxyethyl)amino] propyl]amino]-	363998	513	

680	-	-	603624	483	
681	-	Benzoic acid, 2-hydroxy-, (2,6-pyridinediyldiethylidyne) dihydrazide, nickel	617570	561	
682	-	-	623093	587	
683	-	-	623095	487	
684	-	4-Piperidinone, 3,5-bis[(3,4-dichlorophenyl)methylene]-1-[3-(4-morpholir	638646	591	IVP
685	-	-	640584	516	
686	-	-	640637	513	
687	-	-	640638	537	
688	-	-	640985	478	
689	-	2-Naphthalenebutanamide, N-(3-chloro-1,4-dihydro-1,4-dioxo-2-naphthal	641233	563.95	
690	-	-	641250	507	
691	-	-	642033	477	
692	-	-	642040	526	
693	-	-	644794	536	
694	-	Hexahydroxyethylaminomethyl melamine	653000	565	
695	-	Glycylaminophenylbenzoylurea (HCl salt)	654259	535.78	
696	-	-	657457	547	
697	-	3-(2-chlorobenzylsulfonyl)-3-(2-chlorophenyl)-N-(2,6-dimethylphenyl)-2-ox	657722	490.4	
698	-	Verapamil hydrochloride	657799	491	TOX
699	-	9H-Fluoren-9-one, 2,4,7-trinitro-, (9,10-dihydroanthracen-9-ylidene)hydra	658139	505	
700	-	-	658350	493	
701	-	Naphthalene-1,4-dione, 2-bromo-5,8-dihydroxy-3-[4-[2-(2-hydroxyethoxy)	659999	478	
702	-	5H-Dibenzo[a,d]cyclohepten-5-imine, 10,11-dibromo-10,11-dihydro-N-(b	670140	485	
703	-	Benzoic acid, 2,4-dichloro-, 6-acetyl-1,2,3,4-tetrahydro-4-oxo-1,3-dipheny	671136	567.46	
704	128944-81-0	1-p-Anisyl-3-(N,N-dimethyl-p-aminophenyl)-4,5-bis(p-anisylimino)imidazol	671394	566	
705	-	Benzo[1,2-b:4,3-b']dipyrrole-2,8-dicarboxylic acid, 4,5-dihydro-3-[(4-meth	679524	533	
706	106674-01-5	-	679527	479	
707	-	-	681744	555	
708	-	Spiro[1,3-dithiole-2,7'(3'H)-[1,2]dithiolo[4,3-b]-thiopyran]-4,5,5',6'-tetrac	686349	509	
709	160056-49-5	Adenosine, 2'-amino-2'-deoxy-3',5'-O-[1,1,3,3-tetrakis(1-methylethyl)-1,3-c	687330	509	
710	-	-	690634	519	
711	-	-	699479	562	
712	-	-	703550	492	
713	6377-18-0	Benzo[h][1]benzopyrano[5,4,3-cde][1]benzopyran-5,12-dione, 10-[[6-deoxy	5159	641	
714	124-99-2	.alpha.-L-Mannopyranoside, scillarenin-3 6-deoxy-4-O-.beta.-D-glucopyran	7525	693	IVP TOX
715	33279-57-1	k-Strophanthoside	7530	872.96	
716	23344-17-4	Streptovaricinoic acid, methyl ester	19990	770	
717	-	p-Dioxane, 2,5-bis[(benzoyloxymercuri)methyl]-	30916	758	TOX
718	-	Phenothiazine, 10-[3-(4-methyl-1-piperazinyl)propyl]-2-(trifluoromethyl)-,	46061	644	
719	6833-84-7	4,13,22,31,37,38,39,40-Octaoxapentacyclo[32.2.1.17,10.116,19.125,28	52141	737	
720	62362-59-8	L-glycero-.beta.-L-gluco-Heptopyranosylamine, 4-deoxy-4-[[[(14-methyl-1-c	65104	622	HTX IVP
721	5373-42-2	6a.alpha.-Aporphine, 9-[[4,5-dimethoxy-.alpha.-((S)-1,2,3,4-tetrahydro-6,7	68075	696.84	IVP TOX
722	1404-15-5	2,6-Epoxy-2H-naphthaceno[1,2-b]oxocin-14-carboxylic acid, 11-[(6-deoxy-	70845	788	IVP RPT TOX
723	11048-97-8	4H-Anthra[1,2-b]pyran-4,7,12-trione, 2-(3,3'-dimethyl[2,2'-bioxiran]-3-yl)-1	70929	747	HTX IVP
724	-	Chelocardin, dodecyl sulfate (salt)	73495	678	
725	1404-20-2	Peliomycin	76455	853	ALK HTX
726	548-40-3	N-Methylocoteamine	93135	609	
727	15401-05-5	Emetine, N-(1-carboxyprolyl)-, benzyl ester	103248	712	
728	-	Emetine, N-(N-carboxy-L-tryptophyl)-, N-tert-butyl ester	109350	767	
729	-	-	116693	663	
730	23666-50-4	Rhodomycin A	136044	701	IVP
731	-	2-Propanol, 1,1'-[(1-methylethylidene)bis(4,1-phenyleneoxy)]bis[3-[(1,1,3,3	145366	671.83	
732	35846-53-8	Alanine, N-acetyl-N-methyl-, 6-ester with 11-chloro-6,21-dihydroxy-12,20-	153858	692	IVP
733	11031-82-6	6,9-Metheno-9H-1,3-dioxino[4,5,6-uv][4]benzazacyclo tricosine-20-carbox	156215	812	
734	-	2-Bromo-.alpha.-ergocryptine monomethanesulfonate	169774	751	
735	57576-44-0	1-Naphthacenecarboxylic acid, 2-ethyl-1,2,3,4,6,11-hexahydro-2,5,7-trihy	208734	812	IVP TOX
736	-	Quinolinium, 1-methyl-4-[[4-[[[4-[(1-methylpyridinium-4-yl)amino]phenyl]	218439	622	
737	-	Cinerubine B, hydrochloride	243023	862	IVP TOX
738	57588-03-1	Guanosine, 6-thio-, platinum complex	248436	654	
739	63710-10-1	1-Naphthacenecarboxylic acid, 4-[[O-2,6-dideoxy-.alpha.-L-lyxo-hexopyran	265211	846	IVP TOX
740	-	2,6-Epoxy-2H-naphthaceno[1,2-b]oxocin-9,16-dione, 11-[(6-deoxy 3-C-me	265450	729.78	IVP
741	70878-51-2	5,12-Naphthacenedione, 8-acetyl-10-[[3-[bis-(phenylmethyl)amino]-2,3,6-t	268242	744	IVP
742	-	THALICARPINE TRANSFORMATION PRODUCT	274893	738	
743	-	Ethanol, 2,2'-[[4-[[[3-chloro-4-(2-phenyl-4-thiazolyl)phenyl]imino]methyl]-3	281613	648	ALK
744	63166-73-4	Phyllanthoside	328426	805	IVP TOX
745	90996-54-6	4,7,12,18-Tetraoxatetracyclo[15.3.1.03,5.011,13] heneicos-14-ene-6,19-d	332598	625.76	IVP
746	83759-46-0	Pregna-1,4-diene-3,11,20-trione, 21-[[[[3,6-bis(dimethylamino)-9-acridinyl	337851	696	
747	-	Tin(IV), chlorotriphenyl[1-(4-ethoxyphenyl)-3-cyanoureato]-, hydrogen, tri	353527	692	IVP

748	-	-	359463	644	
749	-	-	600681	695	IVP
750	-	-	607316	827	
751	-	Mn(II) 3,5-diiodosalicylate	619907	833	
752	-	Antineoplaston AS2-1	620261	809	
753	-	Allyl 2,3,4-tri-O-benzyl-6-O-(tert-butyldimethylsilyl)- .alpha.-D-glucopyranc	624953	605	
754	-	-	625331	660	
755	-	-	625873	640	
756	-	2-Propen-1-one, 1-[2-[(3,4-dichlorophenyl)amino]- 4-methyl-5-thiazolyl]-3-	640335	613	
757	-	-	645567	690	
758	-	-	653558	605	
759	-	L-Prolinamide, N,N-dimethyl-L-valyl-L-valyl-N-methyl- L-valyl-L-prolyl-N-(p	669356	677	IVP
760	-	3-Azido-10-[4-(4-(4-benzoylphenyl)methyl)-1-piperazinyl)butyl]phenothiazi	676963	807	
761	17575-20-1	Lanatoside A	7532	969	
762	18378-89-7	Mithramycin	24559	1085.16	IVP RPT TOX
763	6962-42-1	2,2'-Stilbenedisulfonic acid, 4,4'-[(1-hydroxy-4-sulfo- 2-naphthyl)azo]-, tetr	34931	932.78	
764	143-67-9	Vincaleukoblastine sulfate	49842	909.06	IRT IVP RPT TOX
765	7059-24-7	Olivomycin D, 3B-O-(4-O-acetyl-2,6-dideoxy-3-C-methyl-.alpha.-L-arabino-h	58514	1183	HTX IVP
766	2068-78-2	Vincaleukoblastine, 22-oxo-, sulfate	67574	923.04	HTX IRT IVP RPT
767	-	1,3-Naphthalenedisulfonic acid, 6-hydroxy-5-[[4-[[[4-[[4- [(2-hydroxy-3,6-di	76027	1058	
768	-	Vinleurosine sulfate	90636	907	
769	4434-05-3	Pyrrole-2-carboxylic acid, 5-methyl-, diester with 3,3'-[(3-methylpyrrole-2,	107412	1110	
770	-	Polypodine A, mixt. with Polypodine B	115493	977	
771	53123-88-9	Rapamycin	226080	914.18	IVP
772	17904-78-8	Piperidinium europium benzoyl trifluoroacetonate	253995	1093	
773	12677-11-1	Mithramycin, 3(C)-demethyl-4(C)-methyl	269146	1085	HTX IVP
774	50773-42-7	DIOSGENIN TETRAGLYCOSIDE	306864	1015	
775	77327-05-0	77327-05-0L-Tyrosine, N-[1-[N-[4-[[3-hydroxy-4-[[N-[N-[1-(2-hydroxy-1-oxo	325319	1112	HTX IVP
776	-	Kijanimicin, 3(B)-O-de(2,6-dideoxy-.alpha.-L-ribo-hexopyranosyl)-	363182	1187	
777	-	Imidazo[1,2-a]pyridinium, 2,2'-[1,4-phenylenebis(carbonyl- 2-hydrazinyl-1-	603169	1027	
778	-	4,4'-Bis(8-hydroxy-5-sulfo-7-quinolineazo)stilbene-2,2'-disulfonic acid, tetr	638352	934.76	
779	-	-	640342	1020	
780	-	N-acetyl-1-benzyl-muramyl-L-valil-D-isoglutamyl-N-.delta.-butylamino-9'(1	697468	903	
781	38989-37-6	Berbinium, 5,6,7,8,13,13a-hexadehydro-2,3,10,11-tetramethoxy-8-methy	154890	504	
782	-	-	635544	484	
783	-	-	641240	496	
784	-	Benzenebutanamide, N-(3-chloro-1,4-dihydro-1,4-dioxo- 2-naphthalenyl)-.	641245	553	
785	-	-	677392	849	
786	6928-68-3	Diphenyllead diacetate	173046	479	
787	57780-57-1	1,8-Octanediamine, N,N'-di-9-acridinyl-	219734	499	
788	-	4,4'-Bipiperidine, 1,1'-bis[(6-methoxy- 2-naphthalenyl)methyl]-, dihydroch	683792	582	
789	-	[1,2,3,4]tetrathiocino[5,6-d:7,8-d']diimidazole -2,9-dithione, 1,3,8,10-tet	700582	548.91	
790	28643-80-3	Nigericin, monosodium salt	292567	748	IRT RPT TOX
791	-	Vincaleukoblastine, 4'-deoxy-3',4'-epoxy-, 6'-oxide, (3'.alpha.,4'.alpha.)-	304421	825	
792	-	Carbamodithioic acid, dipropyl-, diester with 1,4-piperazinediylbis(2-meth	675593	711	
793	52386-42-2	5-Methyl-tetrahydrohomofolic acid, disodium salt	139490	519	
794	522-51-0	1,10-Decamethylenebis[4-aminoquinaldinium chloride]	166454	528	
795	41992-22-7	2-Aza-8-germaspiro[4.5]decane-2-propanamine, 8,8-diethyl-N,N-dimethyl-,	192965	499	
796	67047-17-0	1,6-Hexanediamine, N,N'-bis(3-methoxy-9-acridinyl)-	260610	531	
797	-	Platinum, bis(2-amino-1,9-dihydro-6H-purine-6-thionato-N7,S6)-	276299	527	
798	477-57-6	Berbaman, 6,6',7,12-tetramethoxy-2,2'-dimethyl-	97338	623	
799	51757-10-9	2,7-(Epoxypentadeca[1,11,13]trienimino)naphtho[2,1-b]furan, rifamycin d	239375	724	
800	65492-82-2	3,5,9-Trioxa-4-phosphaheptacosan-1-aminium, 4-hydroxy-7-methoxy-N,N,	324368	524	
801	50-76-0	Actinomycin D	3053	1255.43	HTX IVP RPT
802	-	Cyclohemimide	18268	3808	ALK CRC IVP TOX
803	-	Thioflavine S	71948	1349	
804	-	Mikamycin, B grade	125176	1393	
805	-	Antlermicin A, monosodium salt	333856	1336.48	
806	-	Polyoxomolybdate phosphate complex, disodium salt	622116	1869	
807	-	-	643834	1172	
808	12656-09-6	Thiostrepton, 1-valine-2-(2,3-didehydroalanine)-	285116	1648.84	PSN
809	11056-06-7	Blenoxane	125066	1512.61	CRC IVP RPT
810	-	-	615593	1365	
811	127-07-1	Hydroxylamine, N-carbamoyl-	32065	76.05	RPT
